# Supplementary material for: Three Months of Strength Training Changes the Gene Expression of Inflammation-Related Genes in PBMC of Older Women: A Randomized Controlled Trial
Source: Cells. 2022 Feb 3;11(3):531. doi: 10.3390/cells11030531 (PMC8834561; doi:10.3390/cells11030531)

Supplementary Figure S1. Pathways affected by 3 months of strength training.

Genes are represented by nodes. Red nodes represent up-regulated genes, green nodes represent down-regulated genes, white nodes were not significantly altered in our study but are part of the knowledge database of Ingenuity Pathways system. The intensity of the color expresses the degree of up- or down-regulation of the genes. The shape of the node represents the functional class of the gene product. For example, a square represents a cytokine, triangle a growth factor... Straight lines with arrows indicate the biological relationship between two nodes while dotted lines represent the indirect interaction between two nodes.

Table of content

Pathways altered in more than 1 intervention group

1A. LXR/RXR Activation in the IST
1B. LXR/RXR Activation in the SET
1.C TREM1 Signaling in the IST
1.D TREM1 Signaling in the SET
1.E TREM1 Signaling in the CON
1.F Cytotoxic T Lymphocyte-mediated Apoptosis of Target Cells in the IST
1.G Cytotoxic T Lymphocyte-mediated Apoptosis of Target Cells in the CON

Pathways only altered after 3 months of IST

1.H IL-7 Signaling Pathway
1.I IL-22 Signaling
1.J 3-phosphoinositide Biosynthesis
1.K GP6 Signaling Pathway
1.L MIF Regulation of Innate Immunity
1.M Toll-like Receptor Signaling
1.N Role of Pattern Recognition Receptors in Recognition of Bacteria and Viruses
1.O Aryl Hydrocarbon Receptor Signaling
1.P Dendritic Cell Maturation

Pathways only altered after 3 months of SET

1.Q Neuroinflammation Signaling Pathway
1.R Osteoarthritis Pathway
1.S ErbB2-ErbB3 Signaling
1.T NRF2-mediated Oxidative Stress Response
1.U Sirtuin Signaling Pathway
1.V OX40 Signaling Pathway

Pathways only altered after 3 months of CON

1.W HMGB1 Signaling
1.X IL-2 Signaling
1.Y GPCR-Mediated Integration of Enteroendocrine Signaling Exemplified by an L Cell
1.Z T Cell Exhaustion Signaling Pathway
1.AA VDR/RXR Activation
1.AB Role of IL-17F in Allergic Inflammatory Airway Diseases
1.AC IL-3 Signaling
1.AD Telomerase Signaling
1.AE Interferon Signaling
1.AF Th2 Pathway
1.AG Wnt/Ca+ pathway
1.AH GPCR-Mediated Nutrient Sensing in Enteroendocrine Cells
1.AI Dopamine-DARPP32 Feedback in cAMP Signaling
1.AJ D-myo-inositol (1,4,5)-Trisphosphate Biosynthesis
1.AK Sphingosine-1-phosphate Signaling

Pathways altered in more than 1 intervention group

<u>LXR/RXR activation pathway</u>

<u>1A. IST (z-score = 2.04)</u>

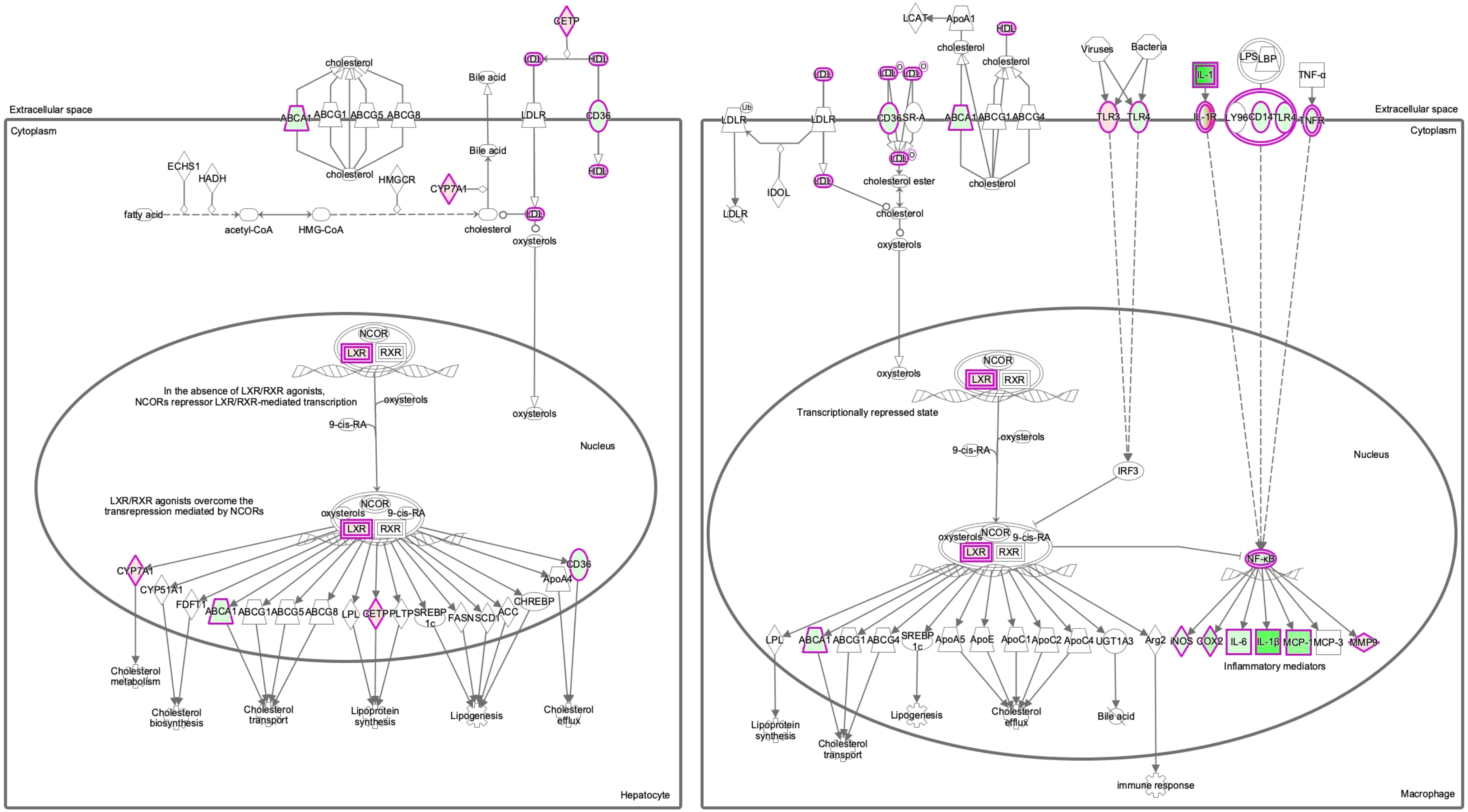

1.B SET (z-score = -2.04)

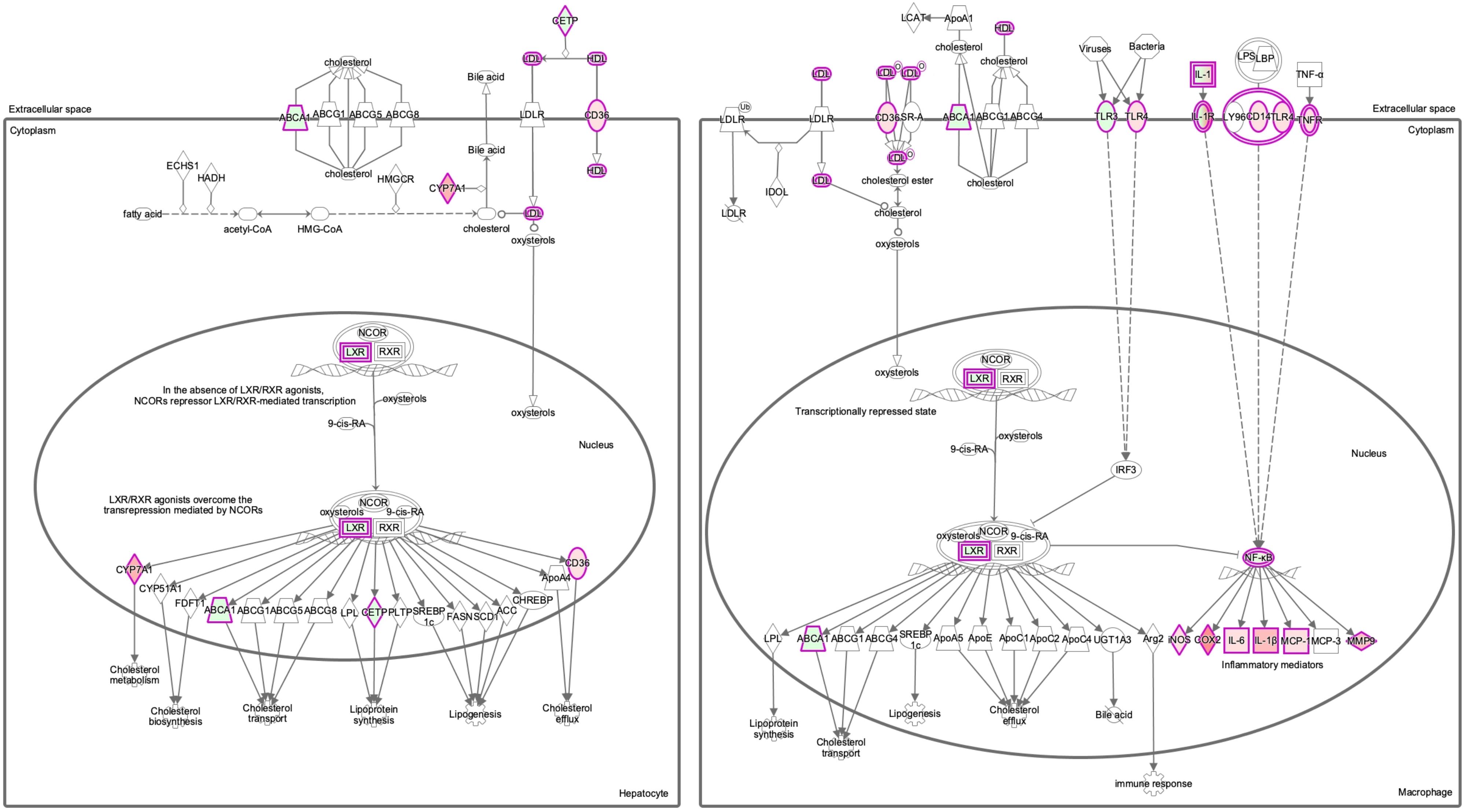

<u>TREM1 signaling</u>

<u>1.C IST (z-score = -2.04)</u>

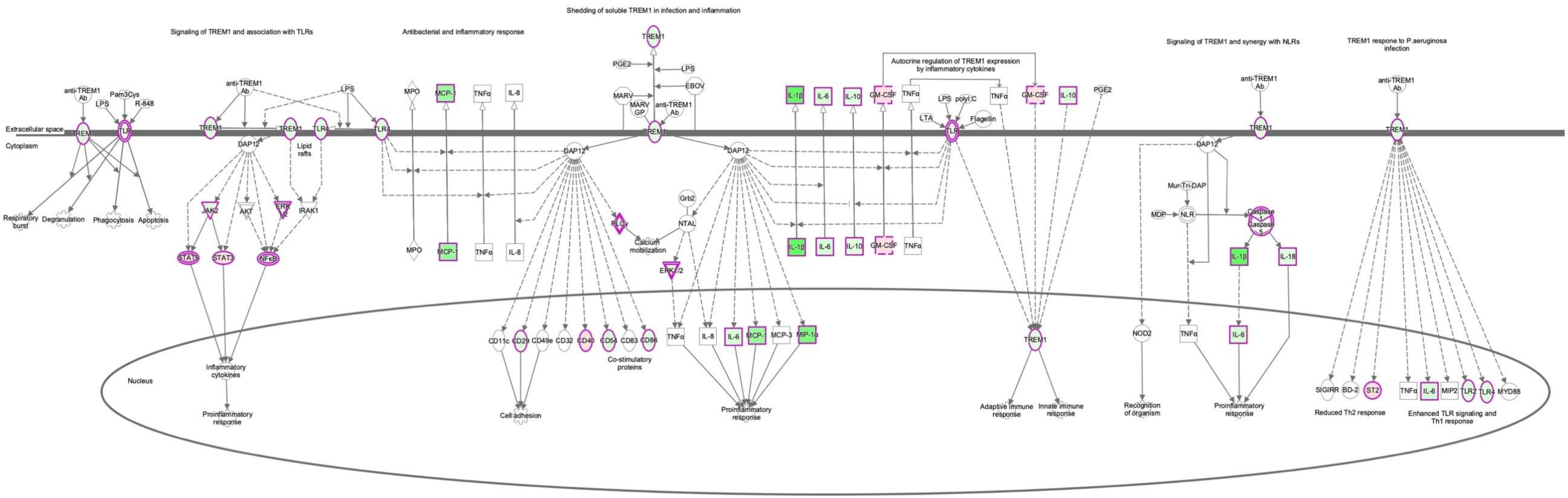

1.D SET (z-score = 2.41)

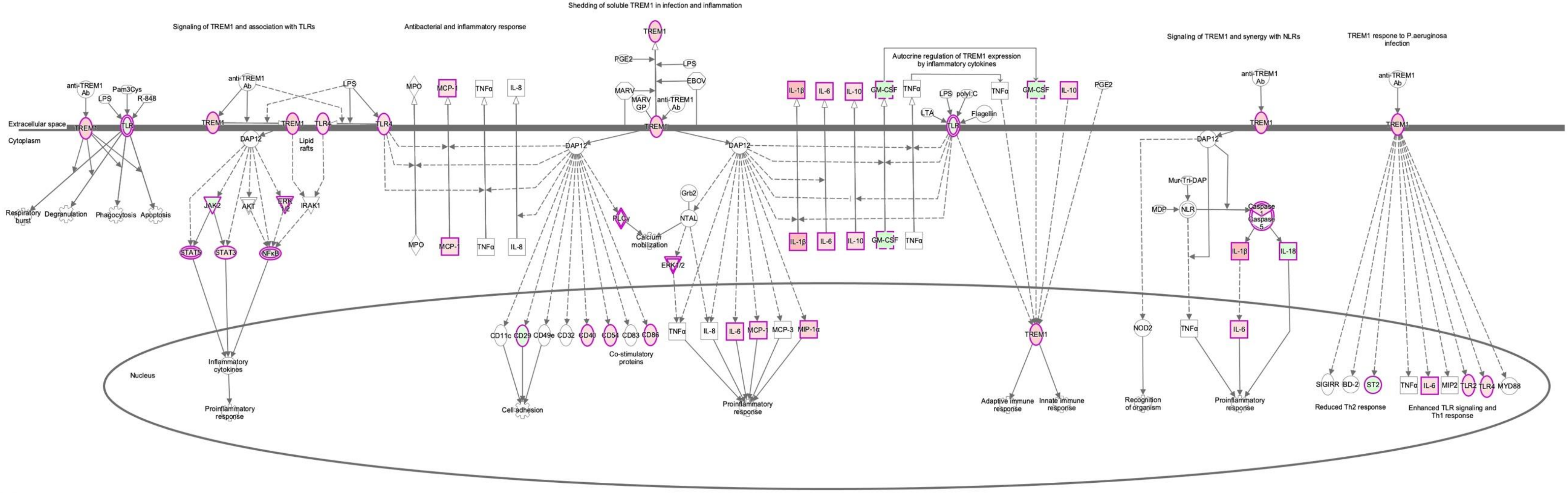

1.E CON (z-score = 2.41)

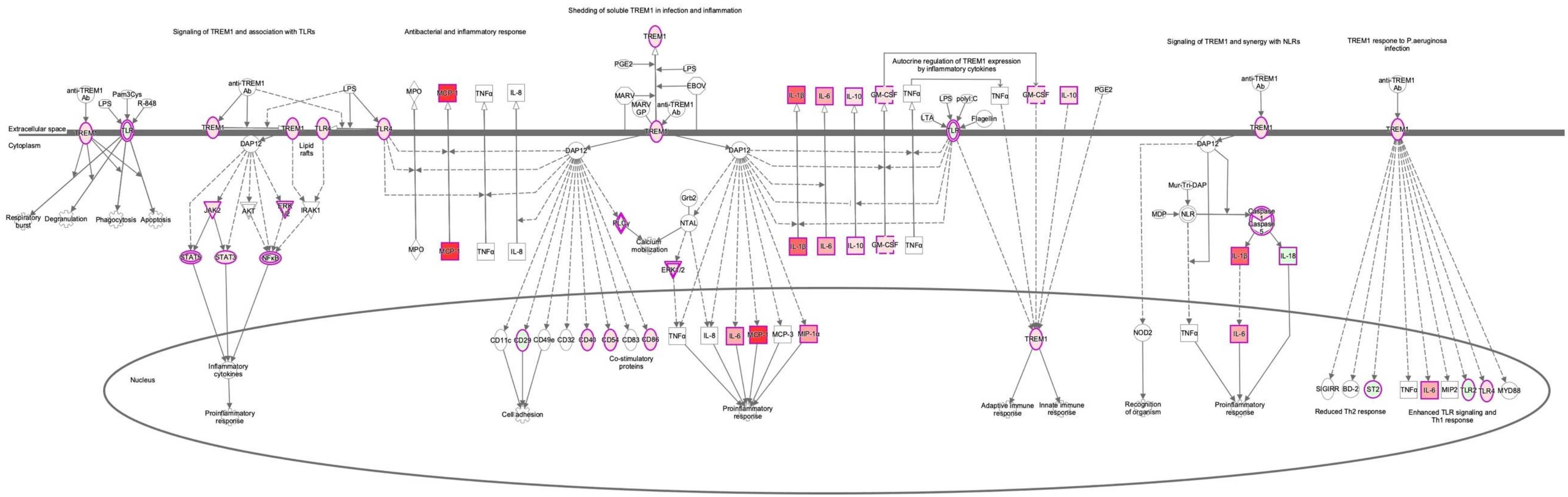

Cytotoxic T lymphocyte-mediated apoptosis of target cells

1.F IST (z-score = 2.12)

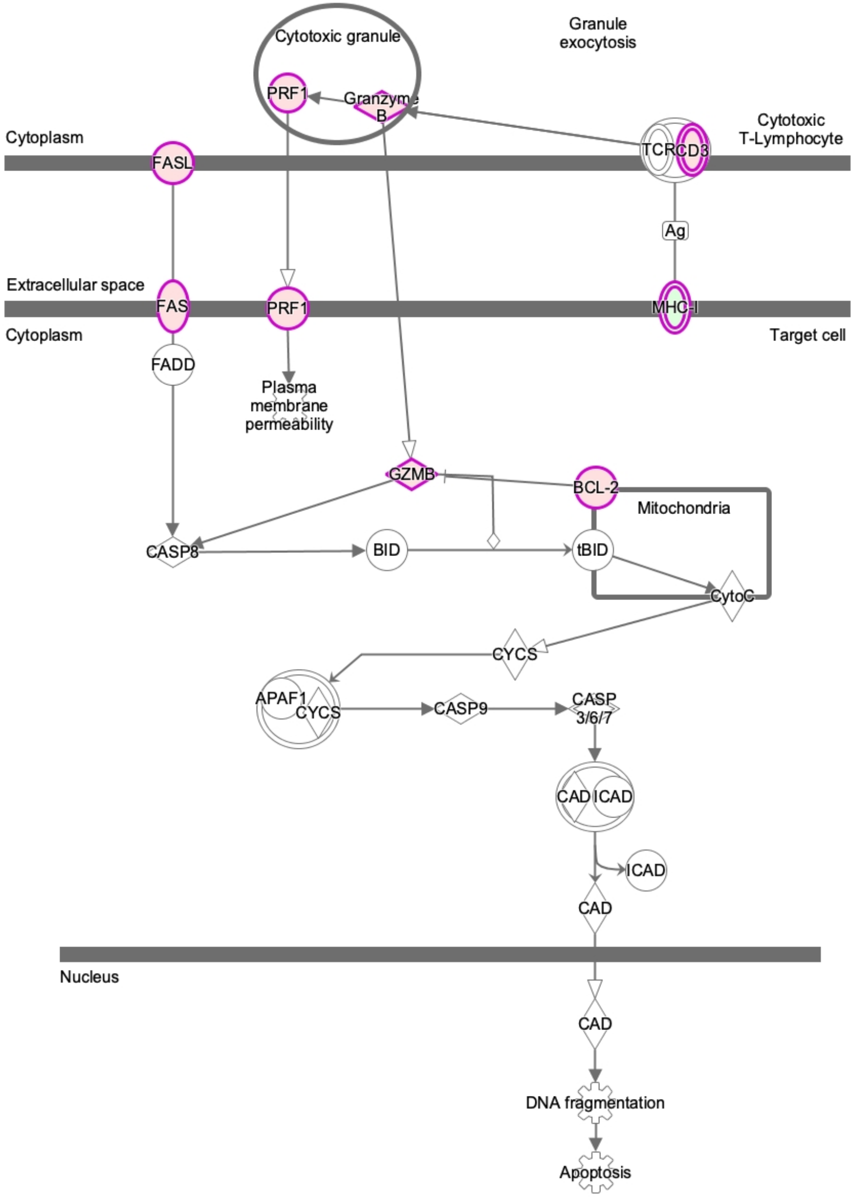

1.G CON (z-score = 2.12)

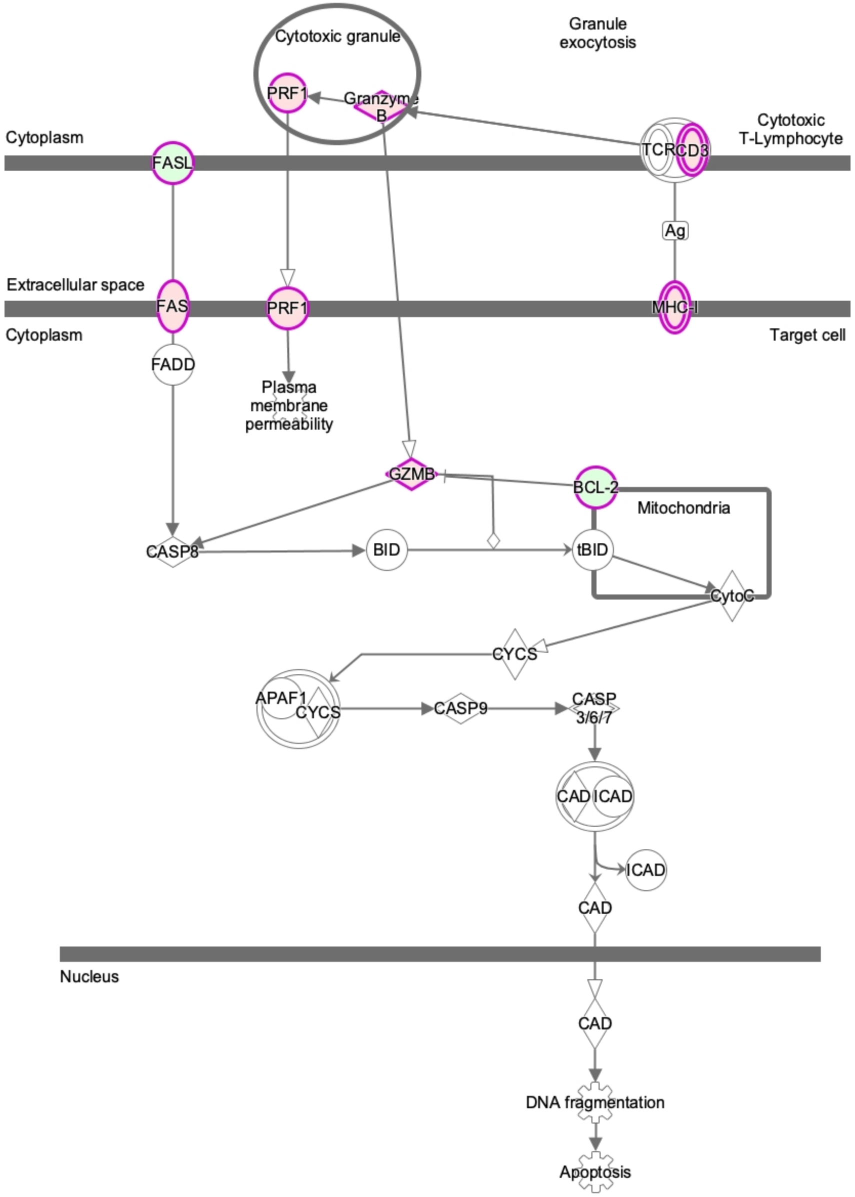

Pathways only altered after 3 months of IST

1.H IL-7 signaling (z-score = 2.14)

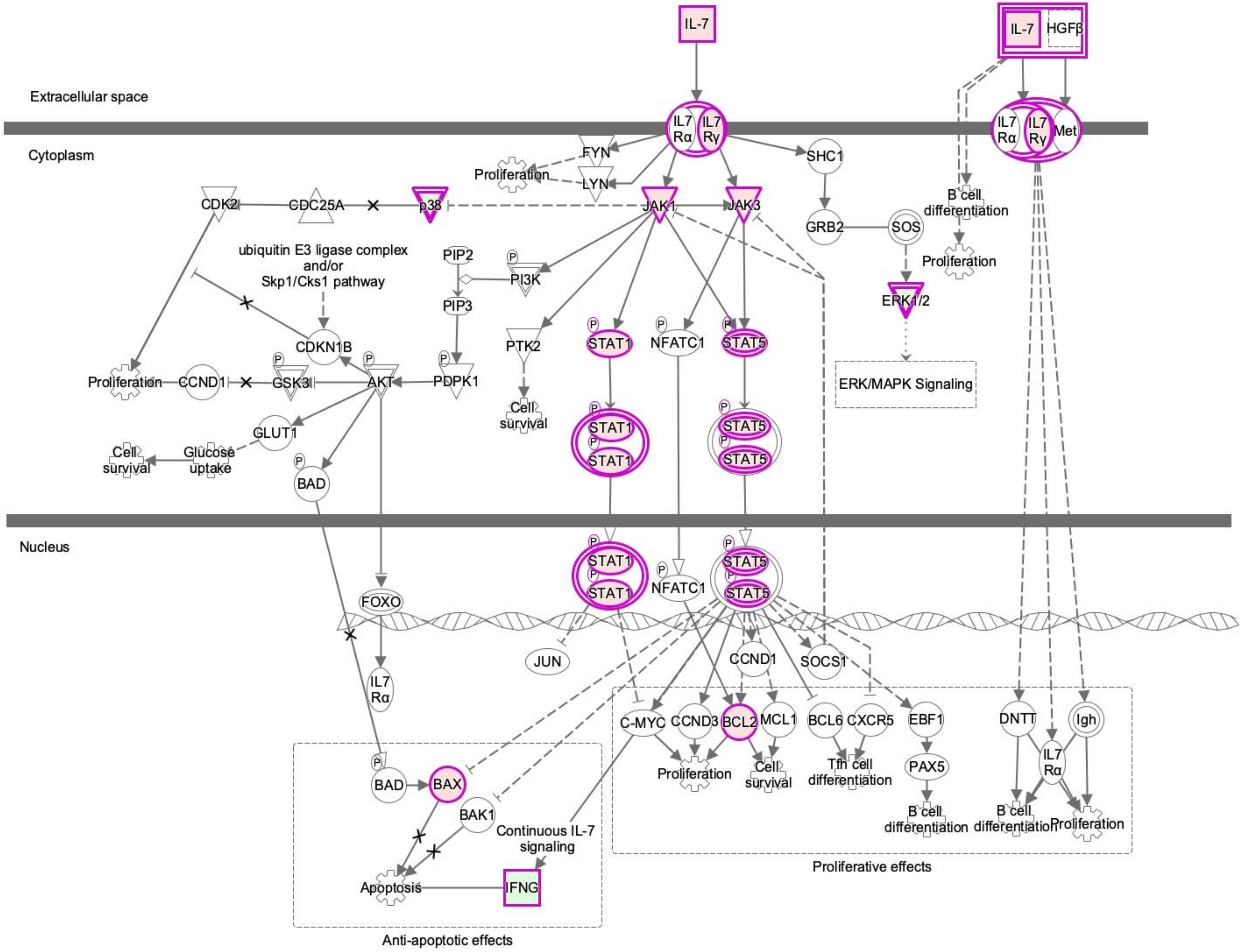

1.I IL-22 signaling (z-score = 2.11)

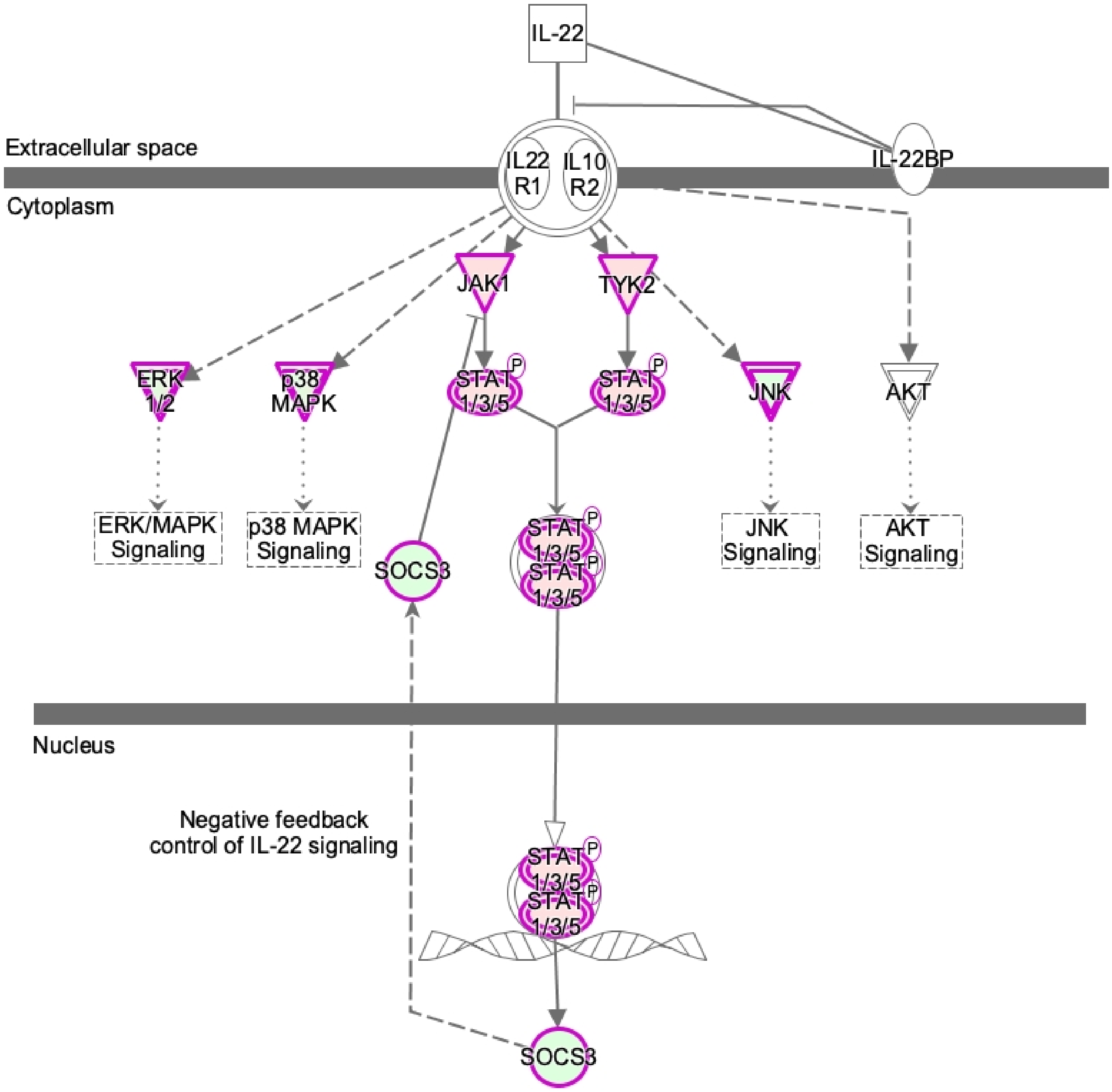

1.J 3-phosphoinositide biosynthese (z-score = -2.11)

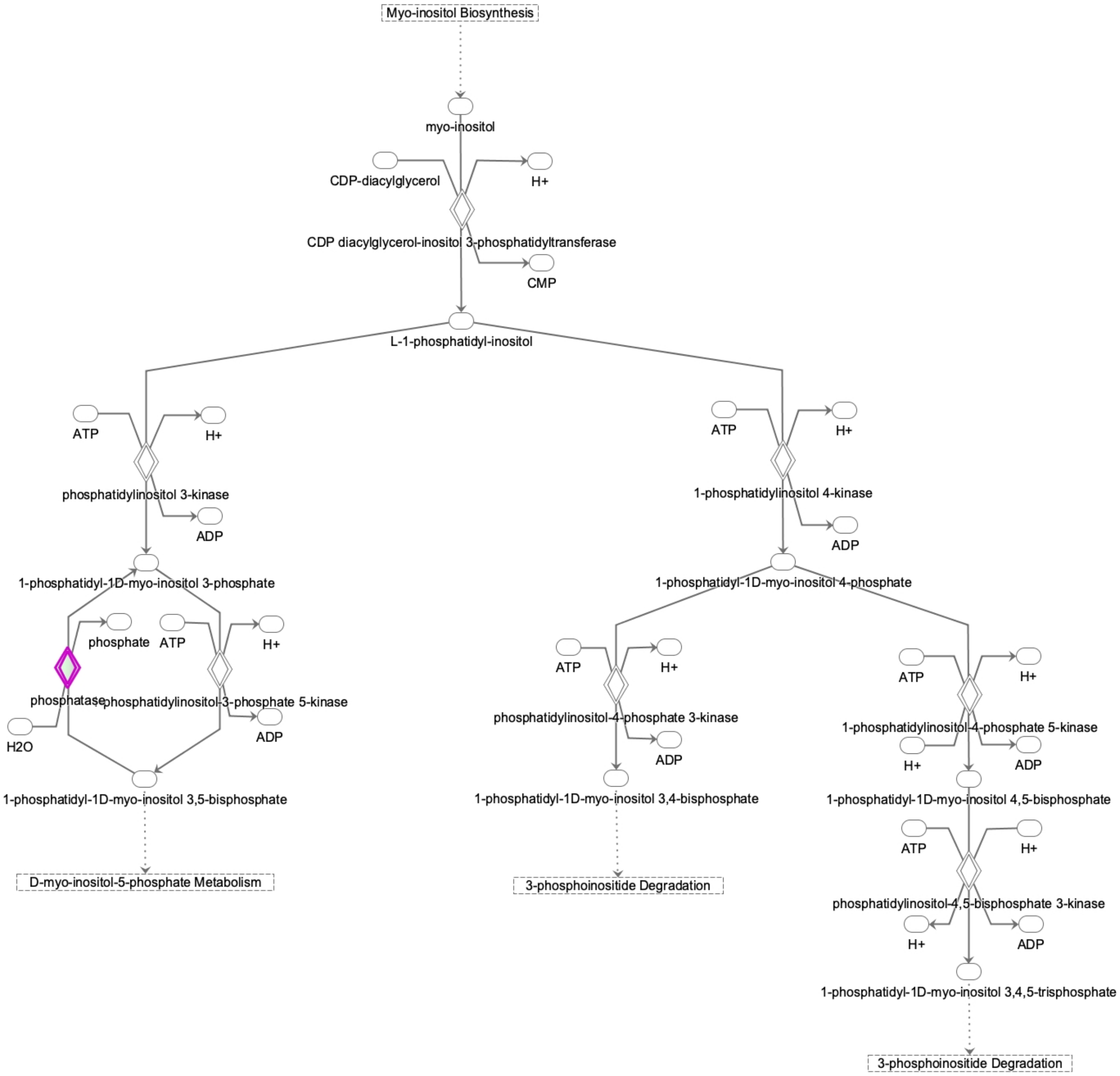

1.K GP6 signaling pathway (z-score = -2.12)

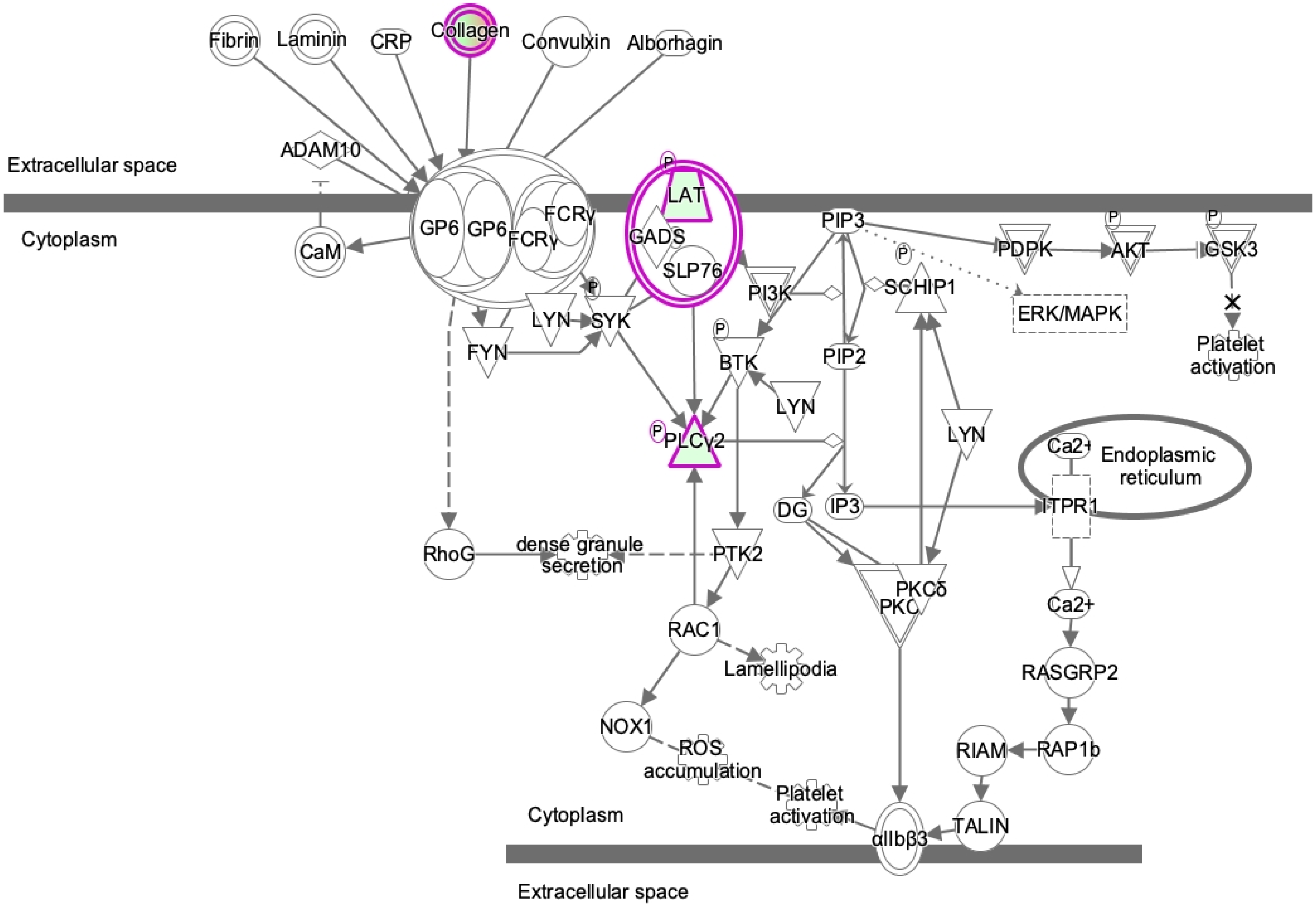

1.L MIF regulation of innate immunity (z-score = -2.14)

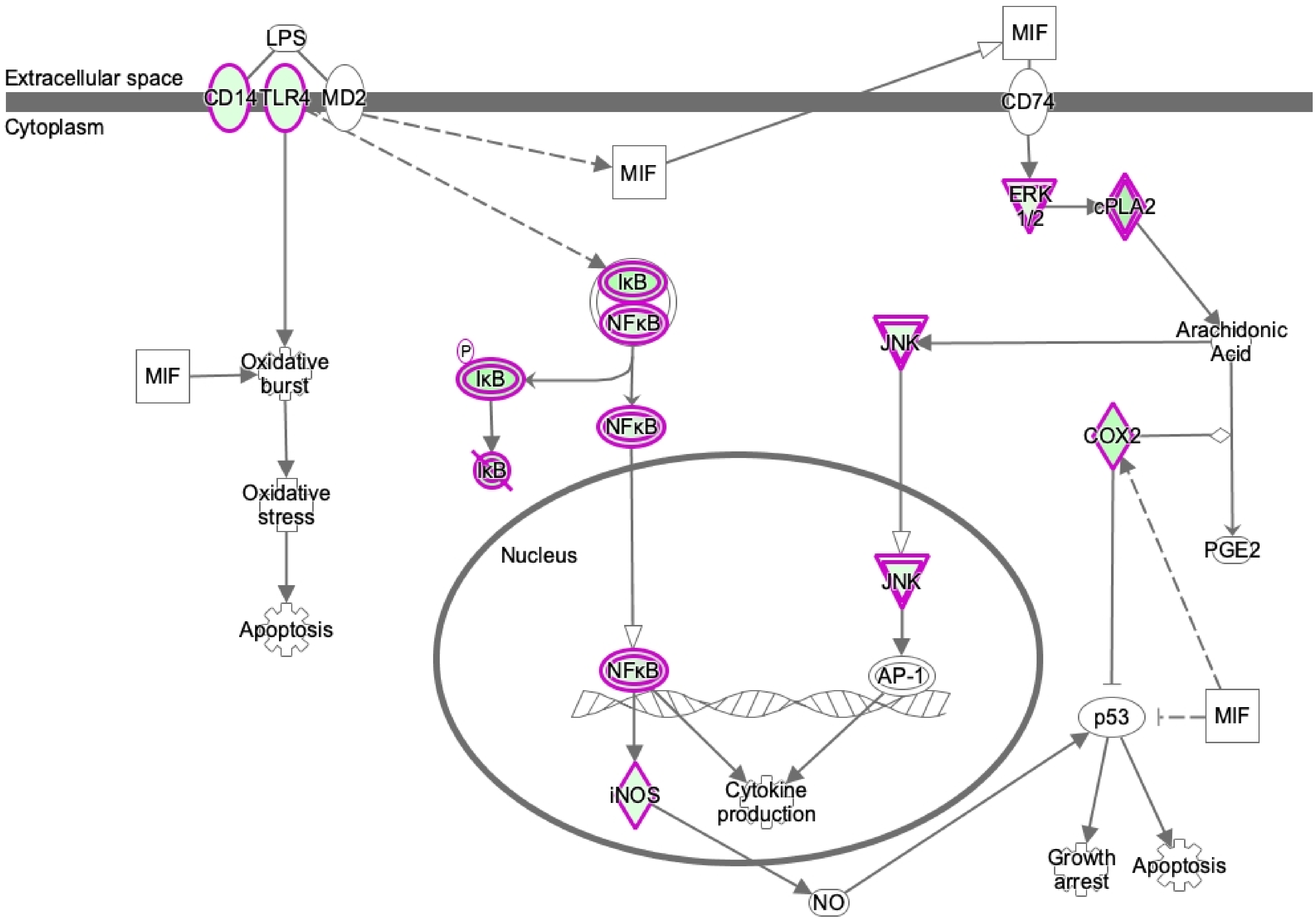

<u>1.M Toll-like receptor signaling (z-score = -2.18)</u>

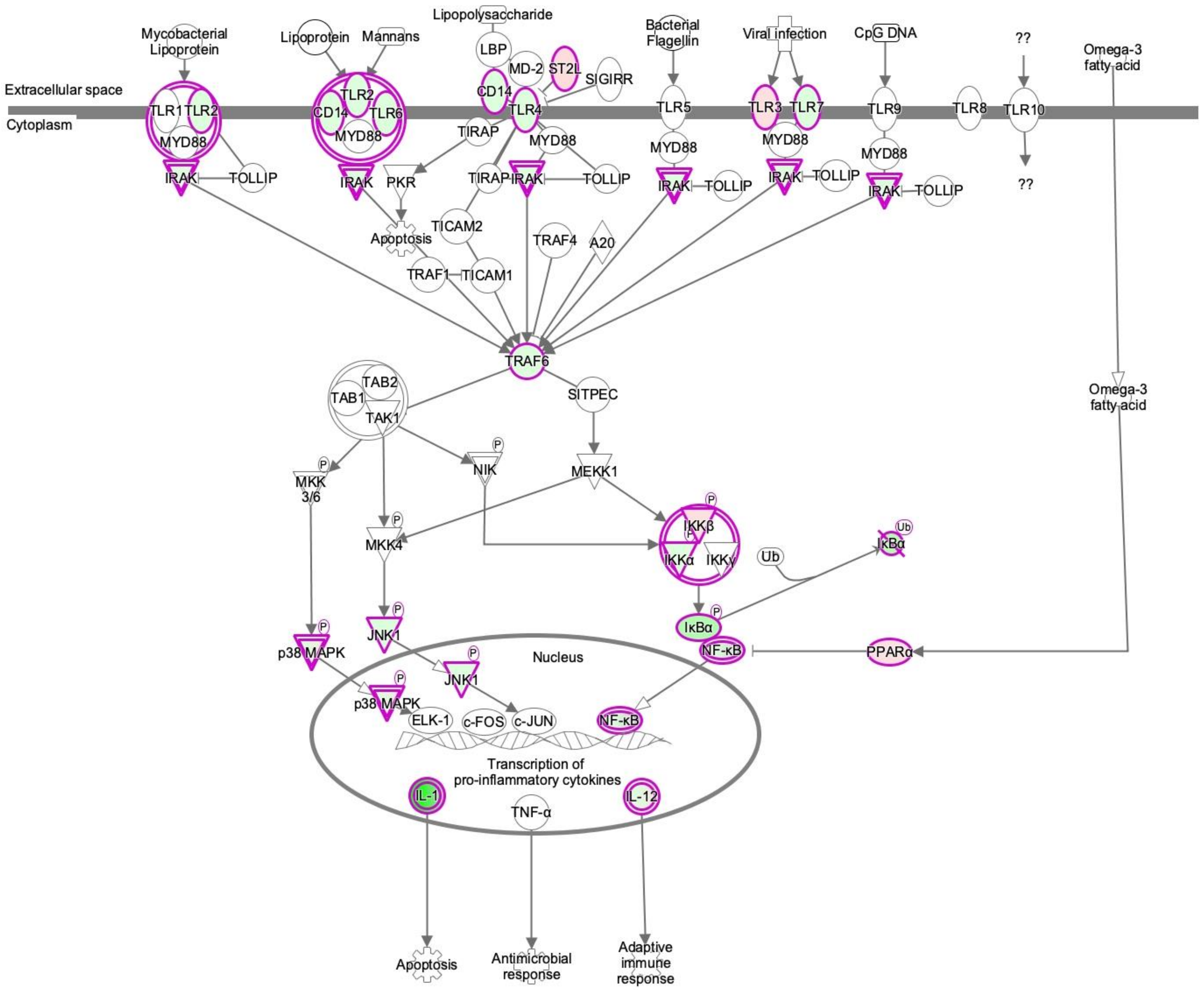

1.N Role of pattern recognition receptors in recognition of bacteria and viruses (z-score = -2.24)

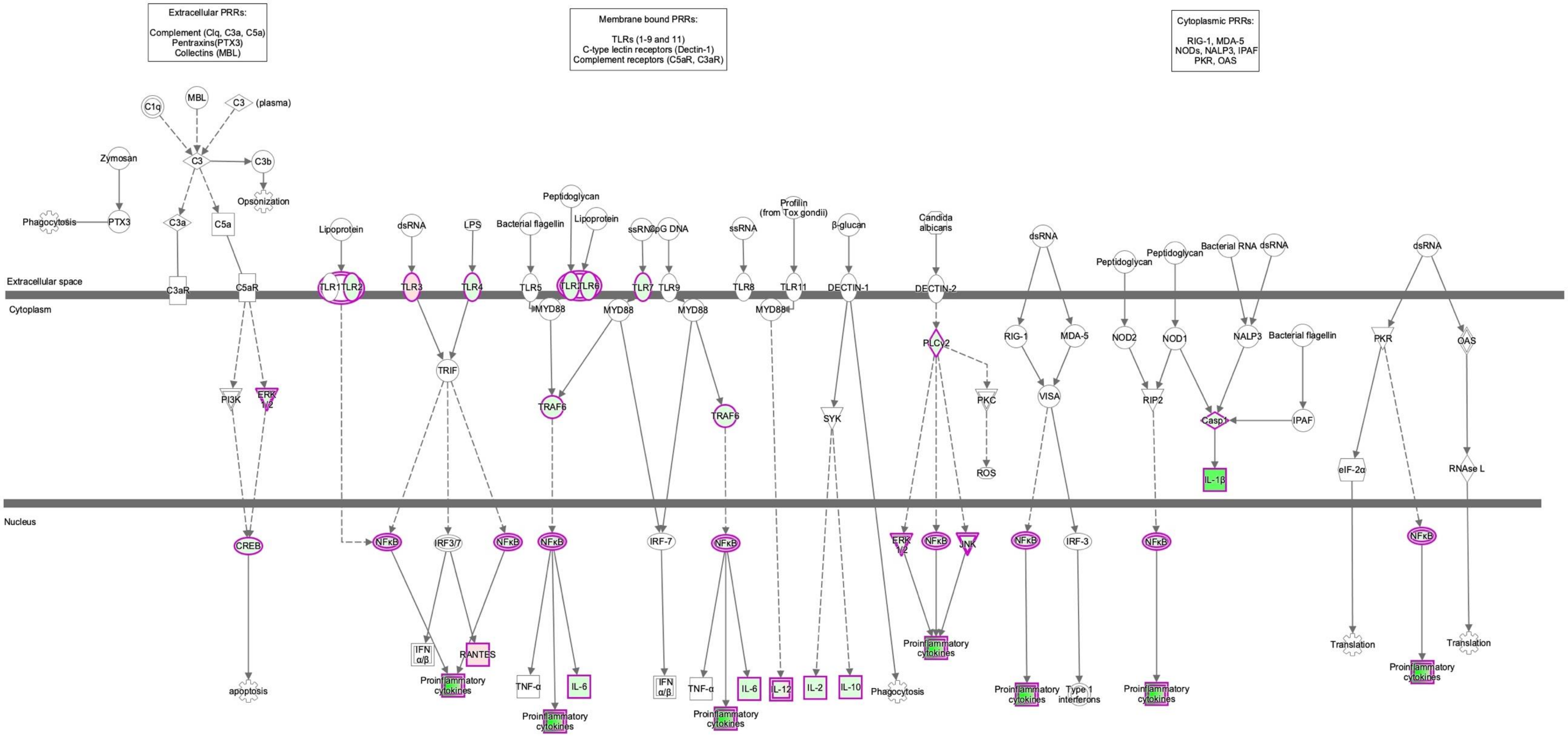

1.O Aryl hydrocarbon receptor signaling (z-score = -2.84)

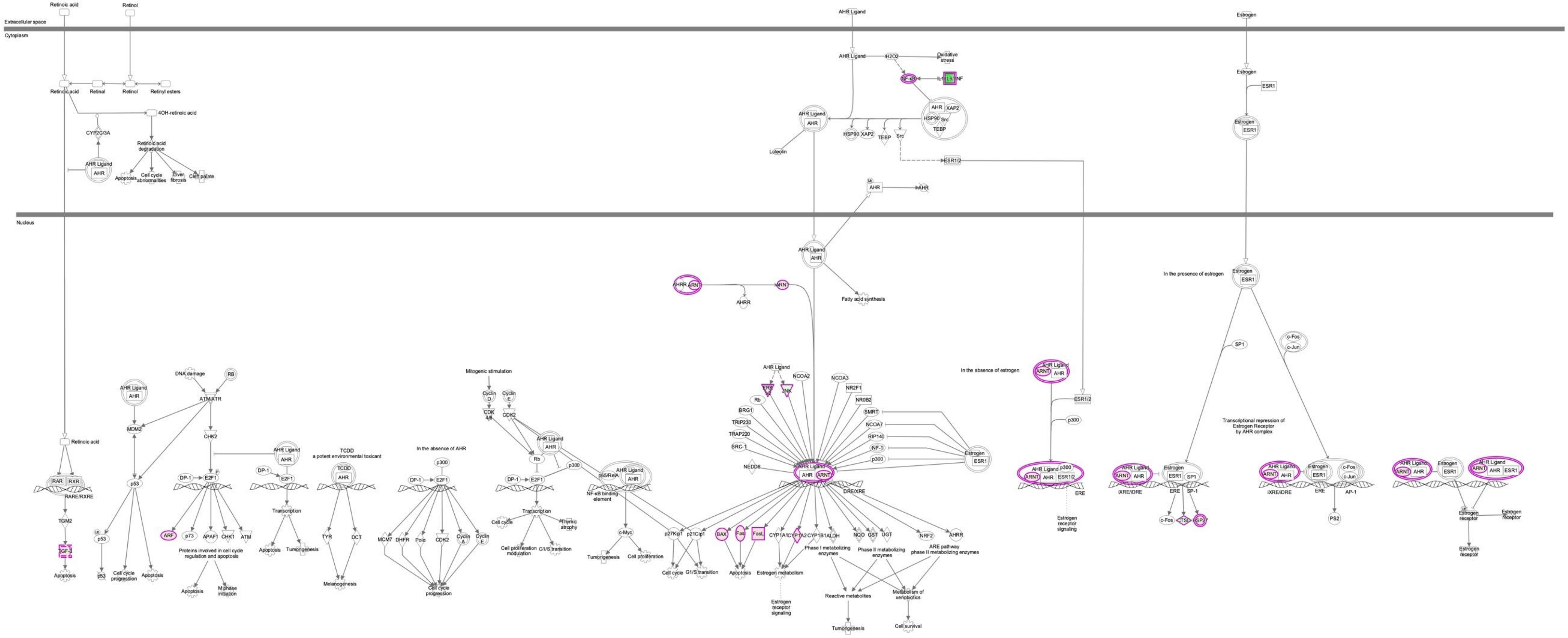

1.P Dendritic cell maturation (z-score = -2.94)

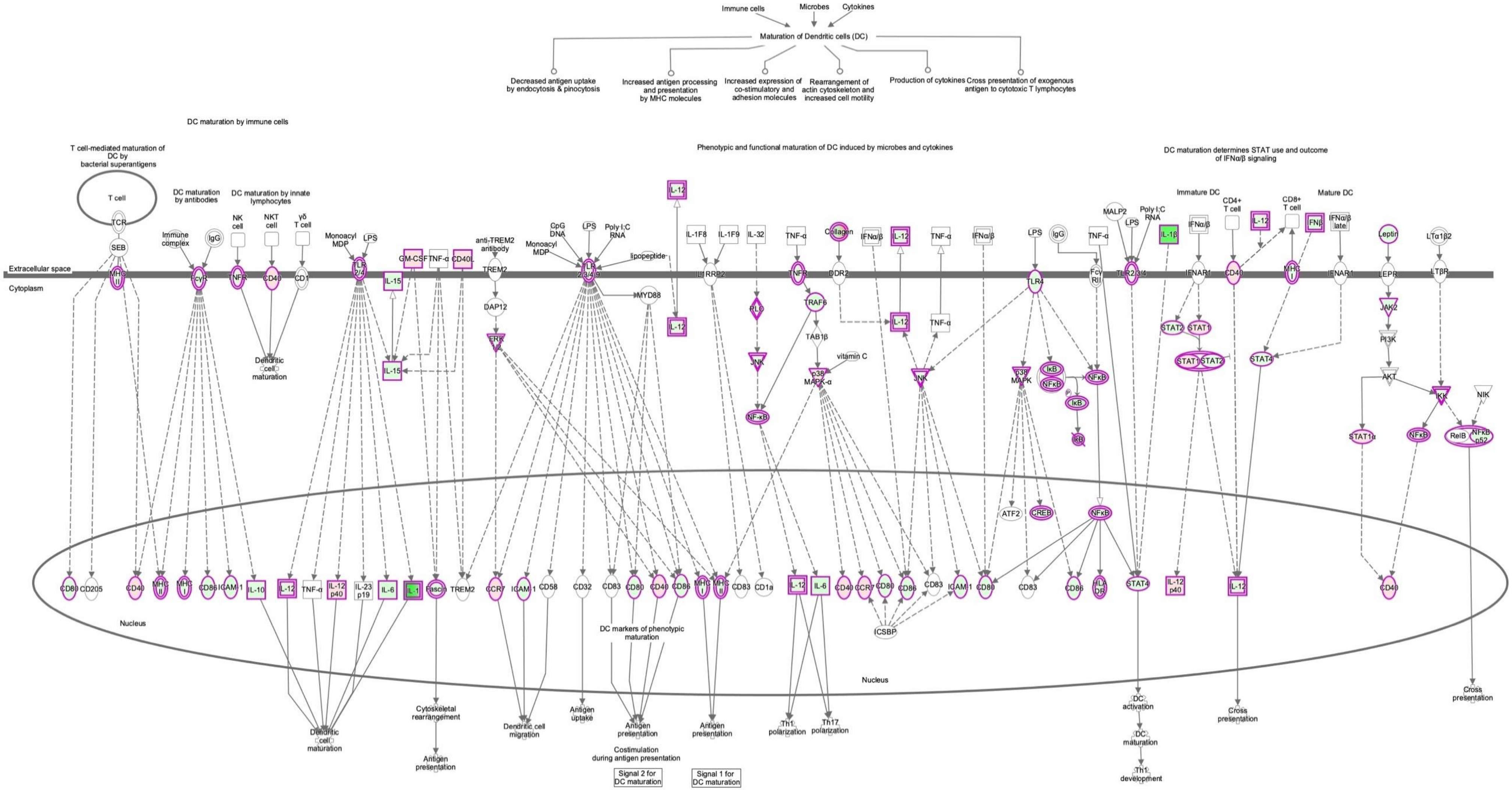

Pathways only altered after 3 months of SET

1.Q Neuroinflammation signaling pathway (z-score = 3.05)

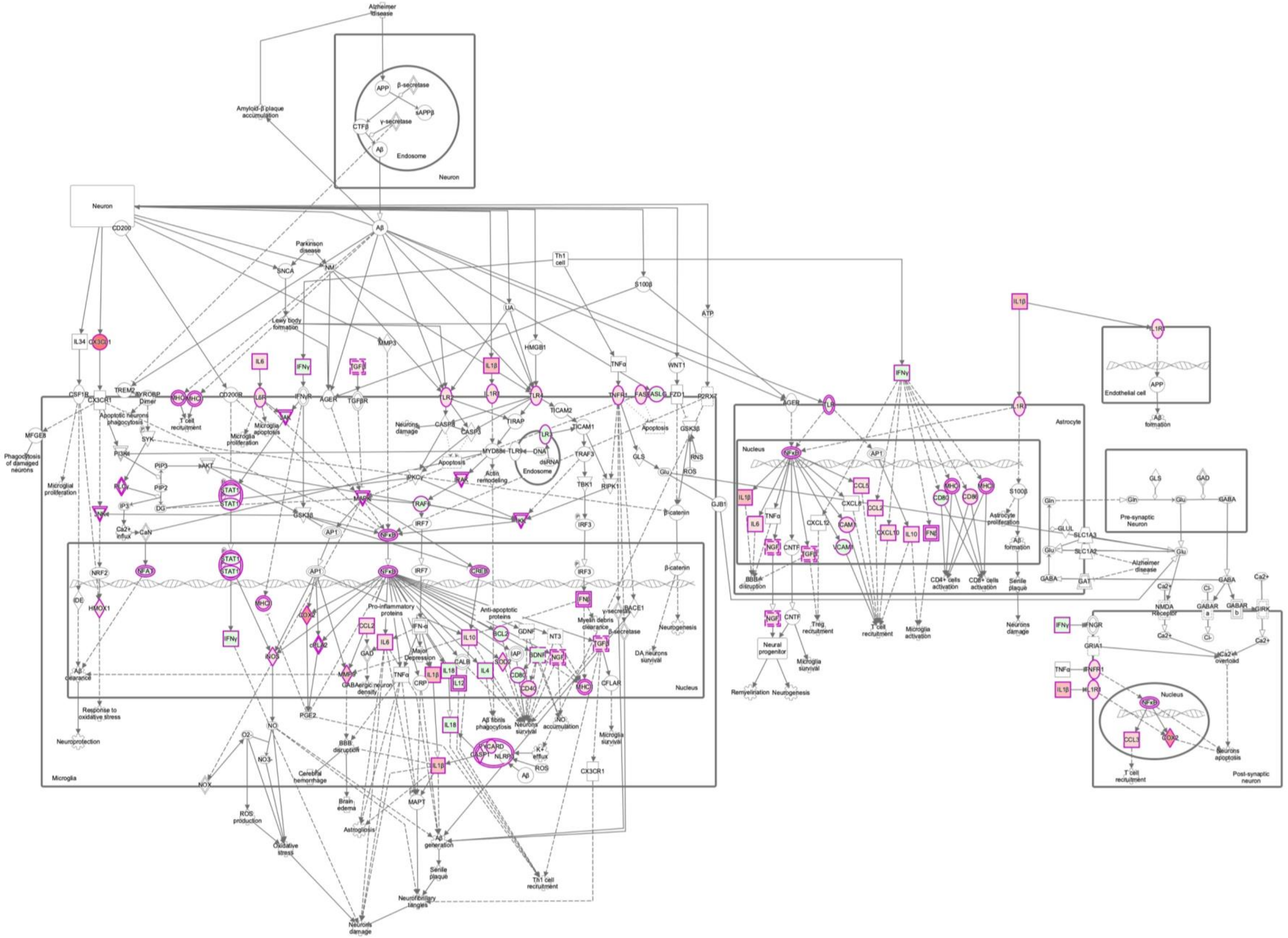

1.R Osteoarthritis pathway (z-score = 2.48)

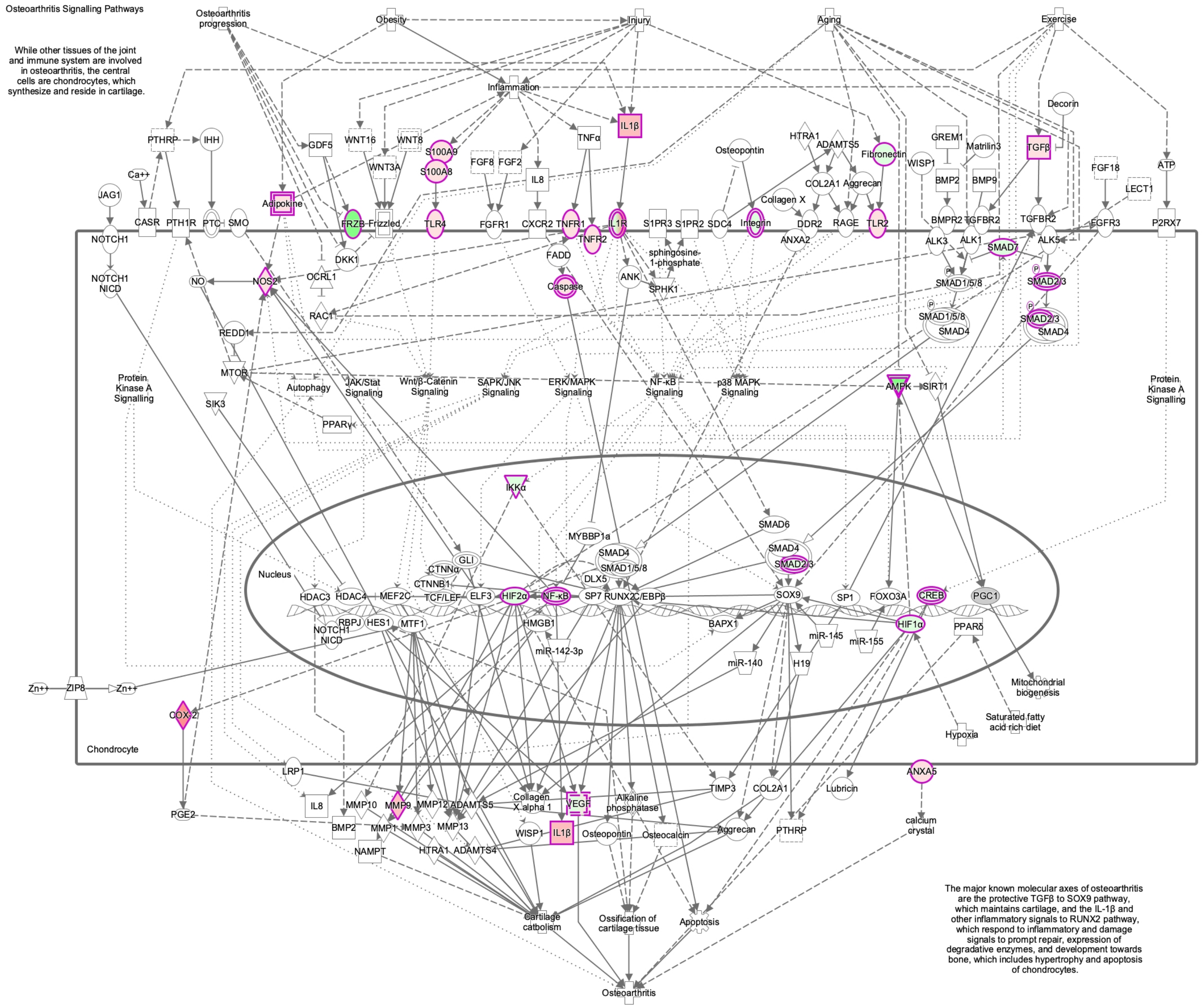

1.S ErbB2-ErbB3 signaling (z-score = 2.33)

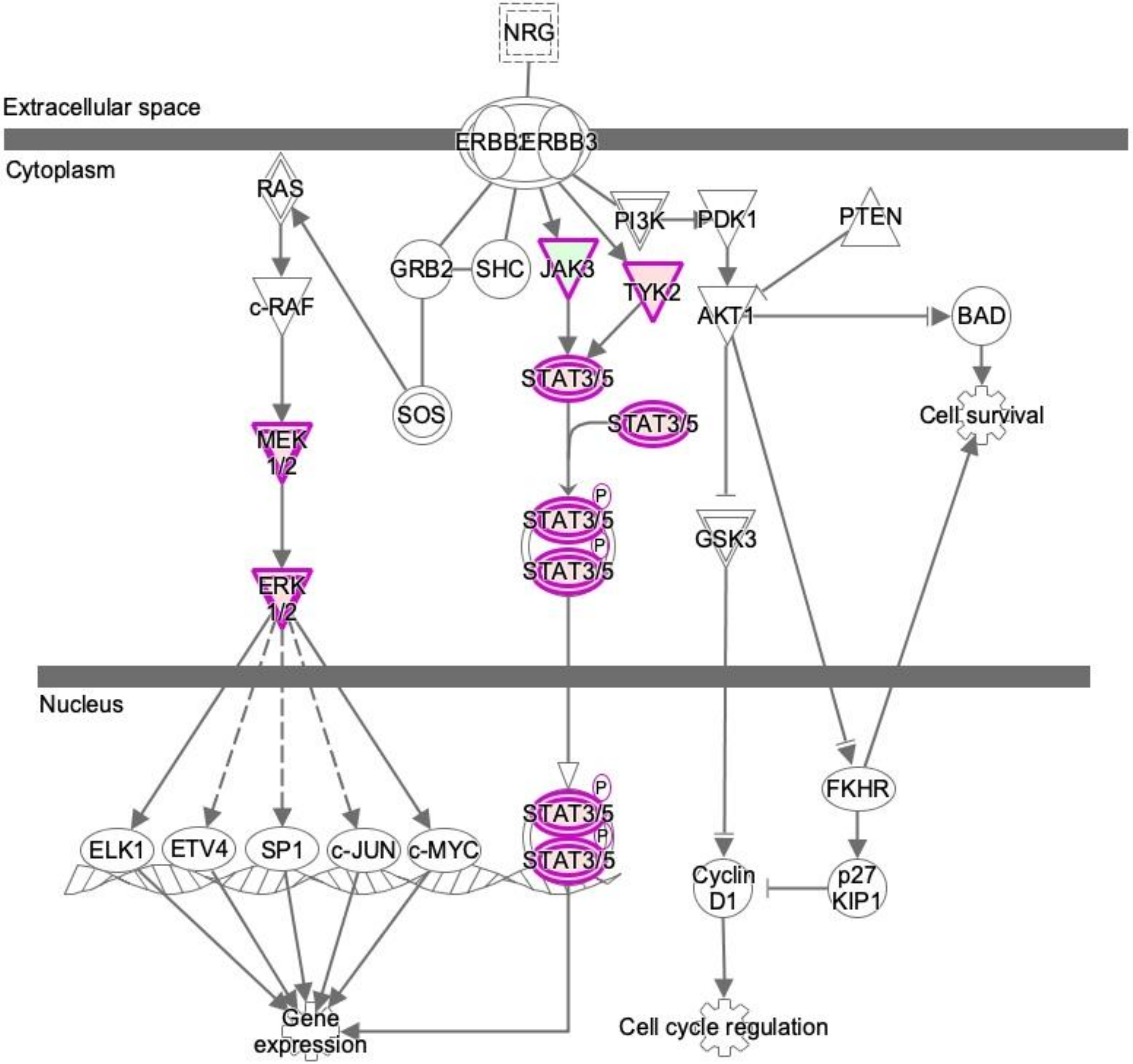

1.T NRF2-mediated oxidative stress response (z-score =2.12)

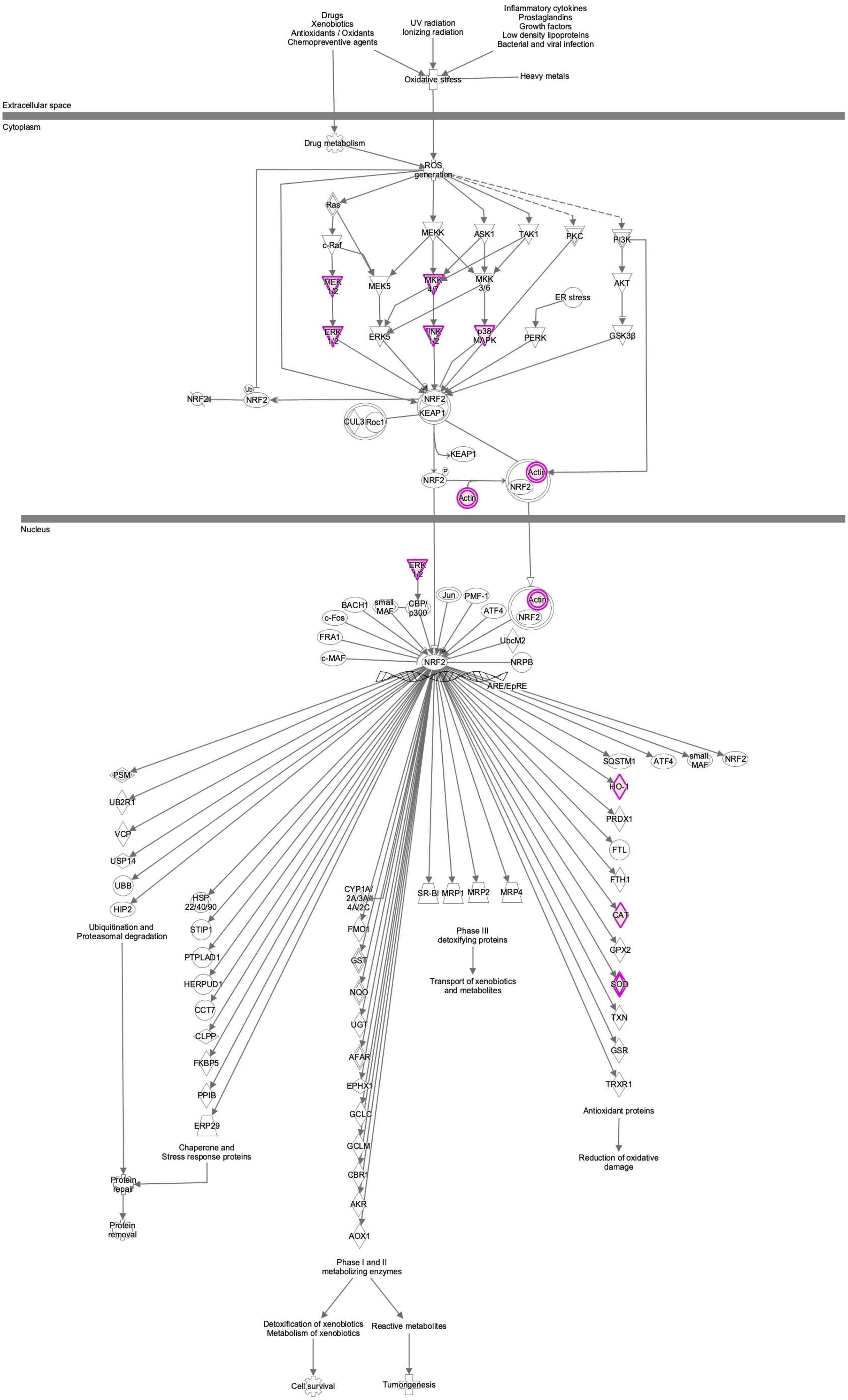

1.U Sirtuin signaling pathway (z-score = -2.52)

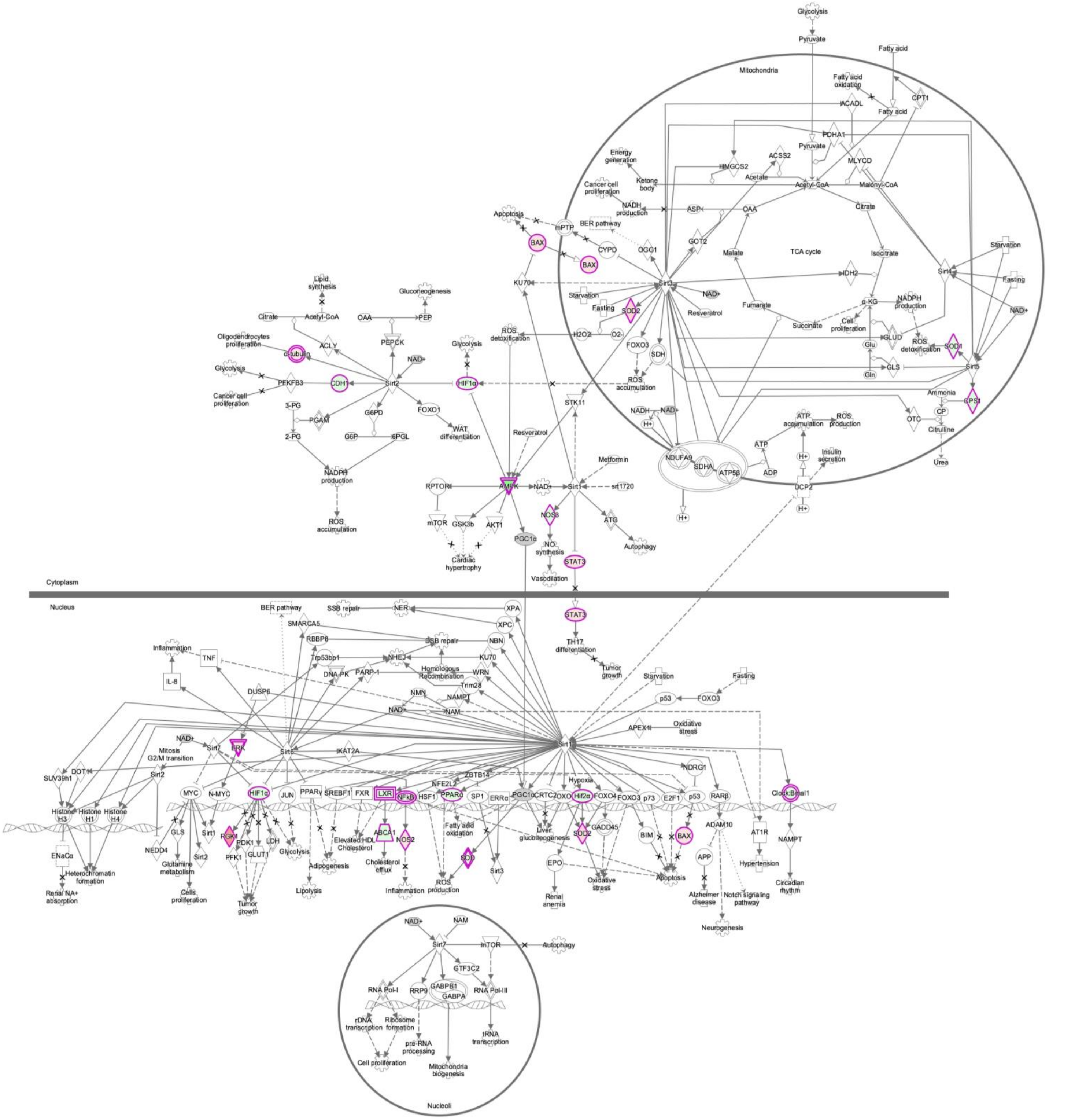

1.V OX40 signaling pathway (z-score = -2.71)

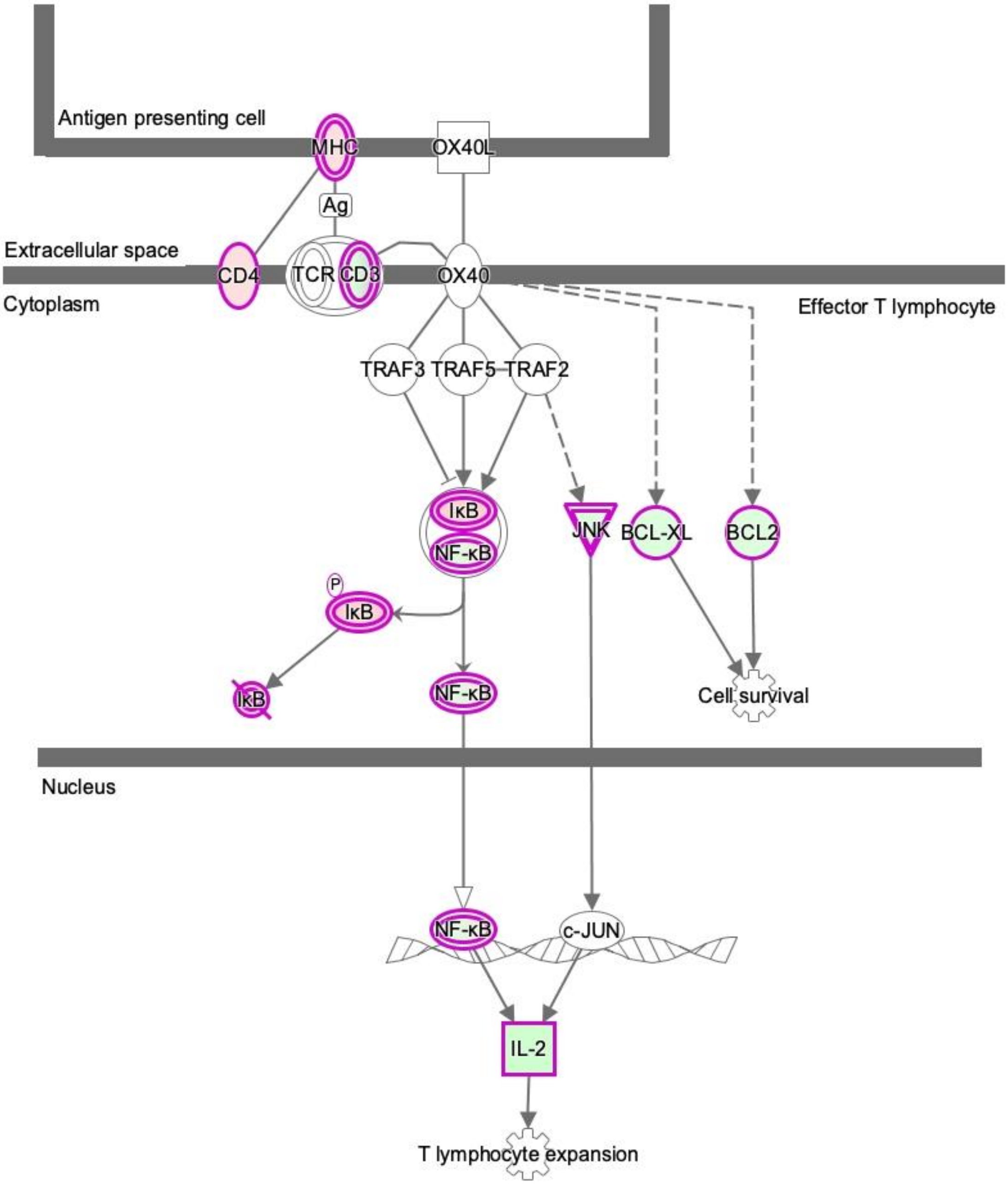

Pathways only altered after 3 months of CON

1.W HMGB1 signaling (z-score = 3.05)

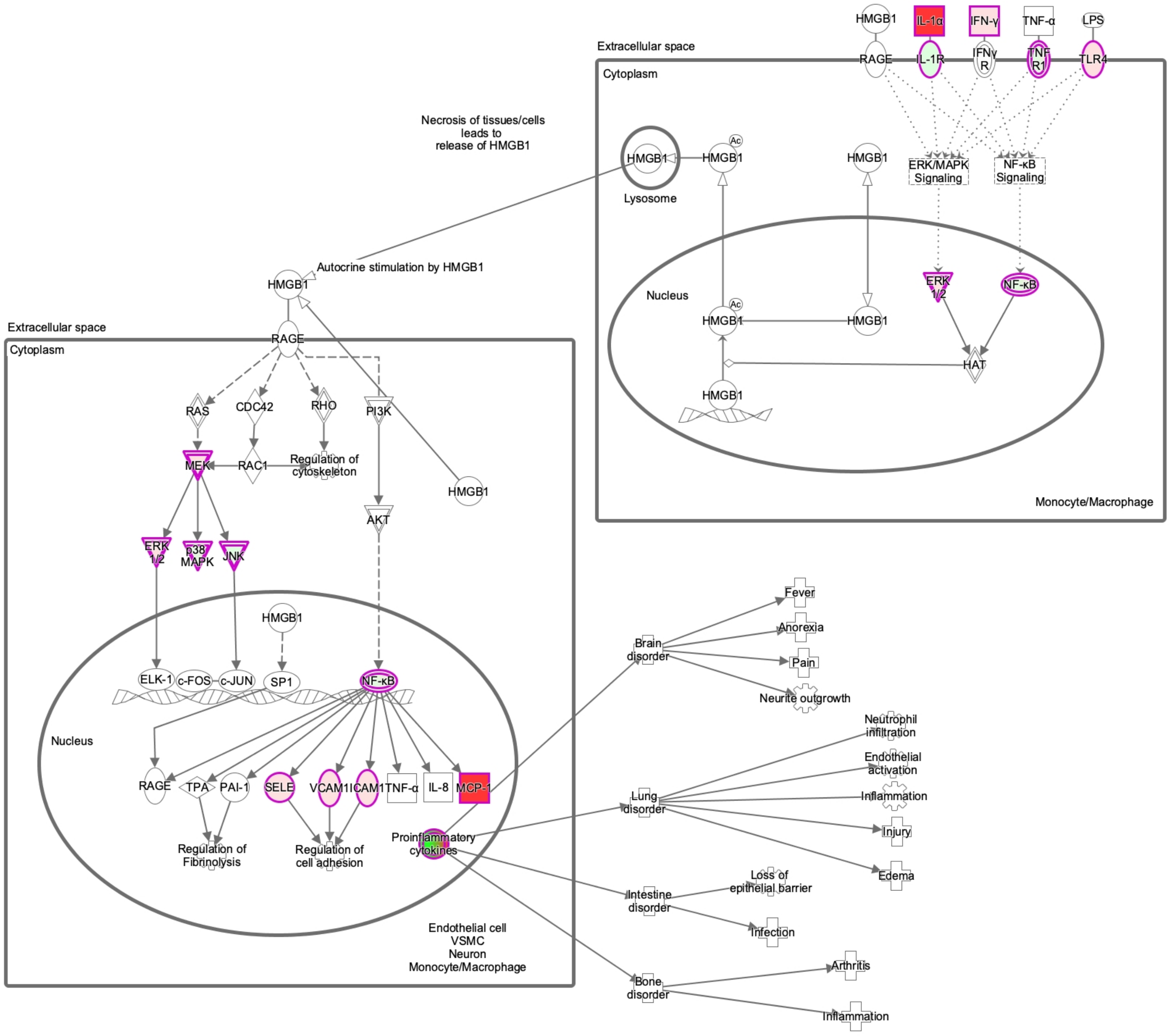

1.X IL2 signaling (z-score = 3.05)

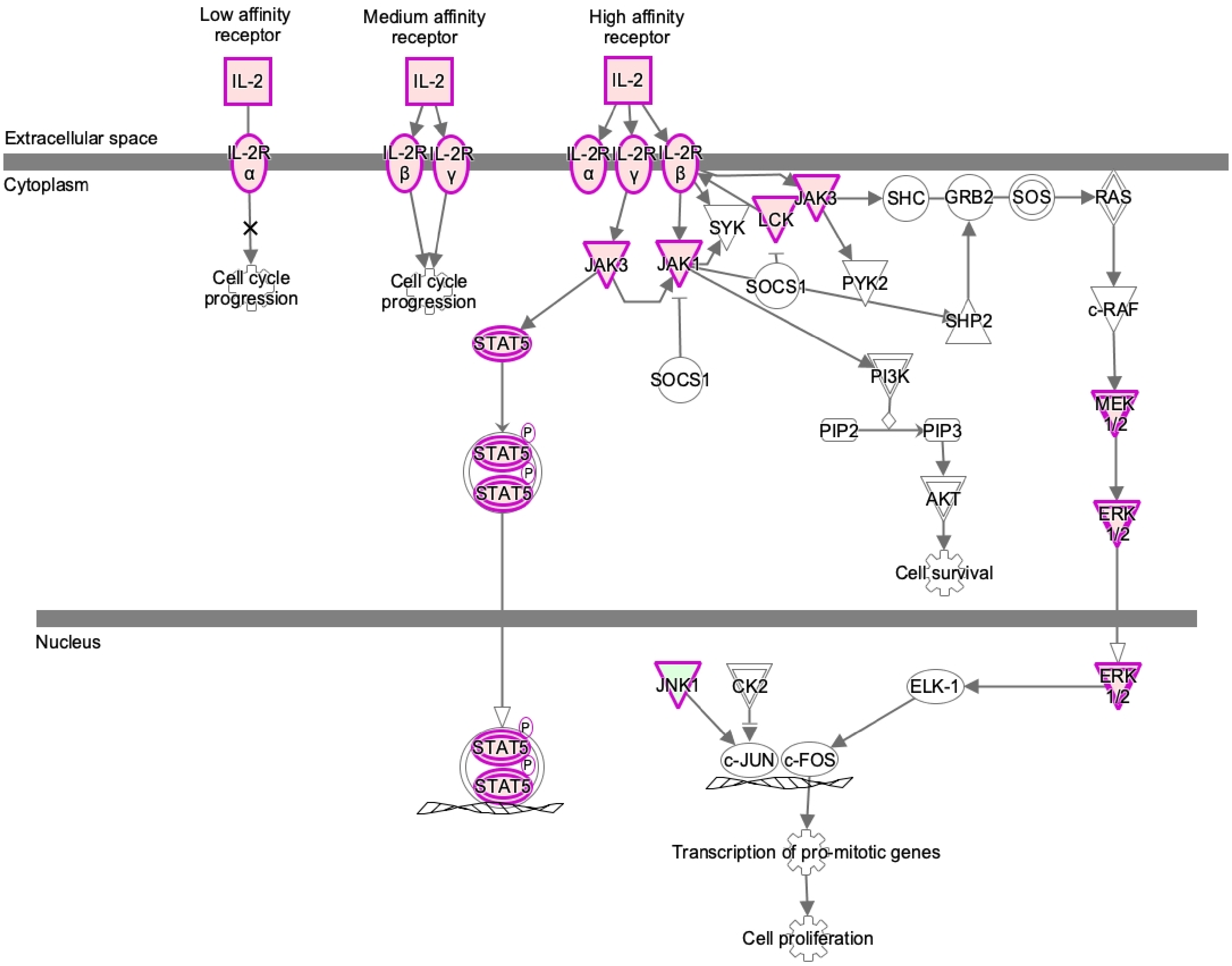

1.Y GPCR-mediated integration of enteroendocrine signaling exemplified by an L cell (z-score = 3.00)

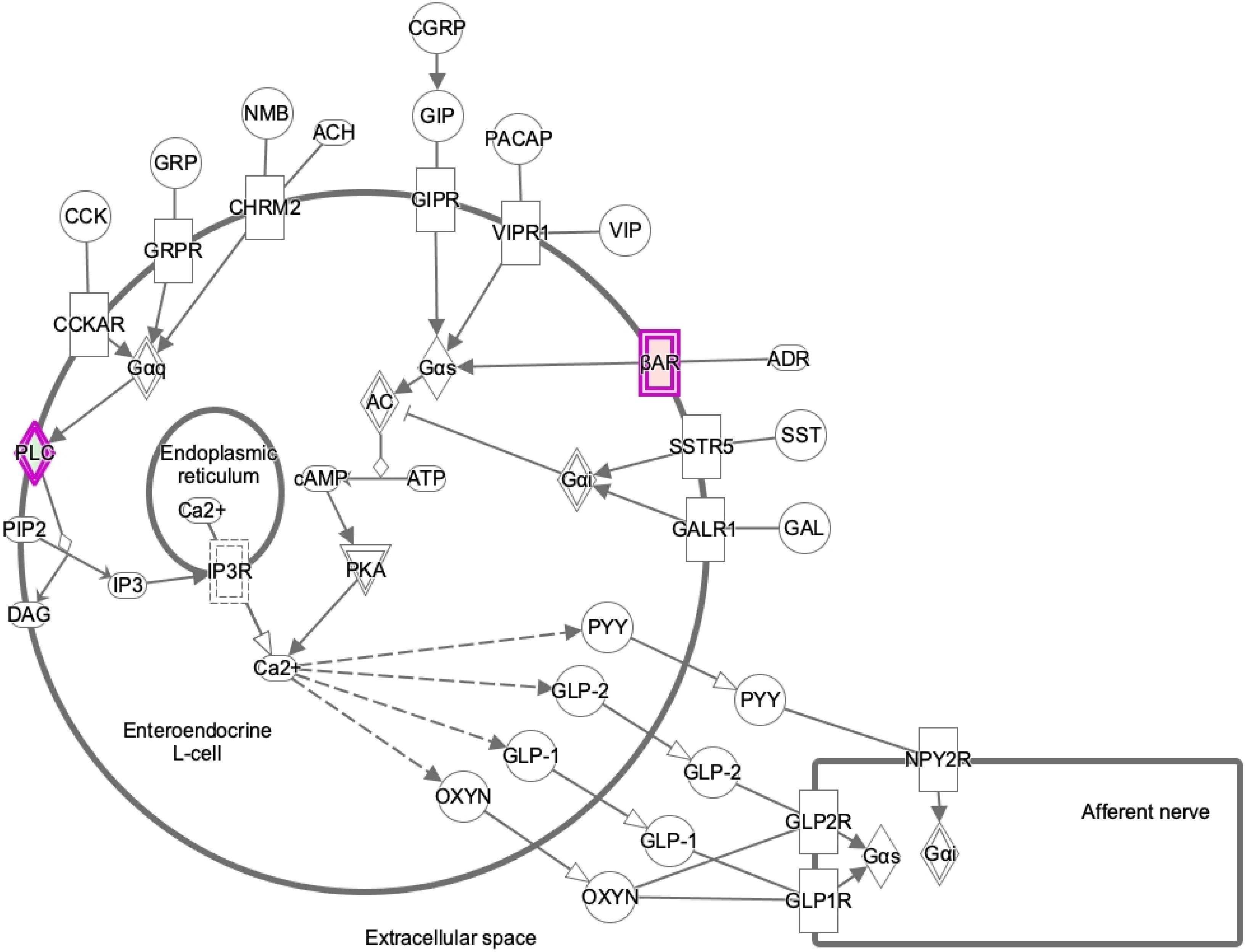

1.Z T cell exhaustion signaling pathway (z-score = 2.65)

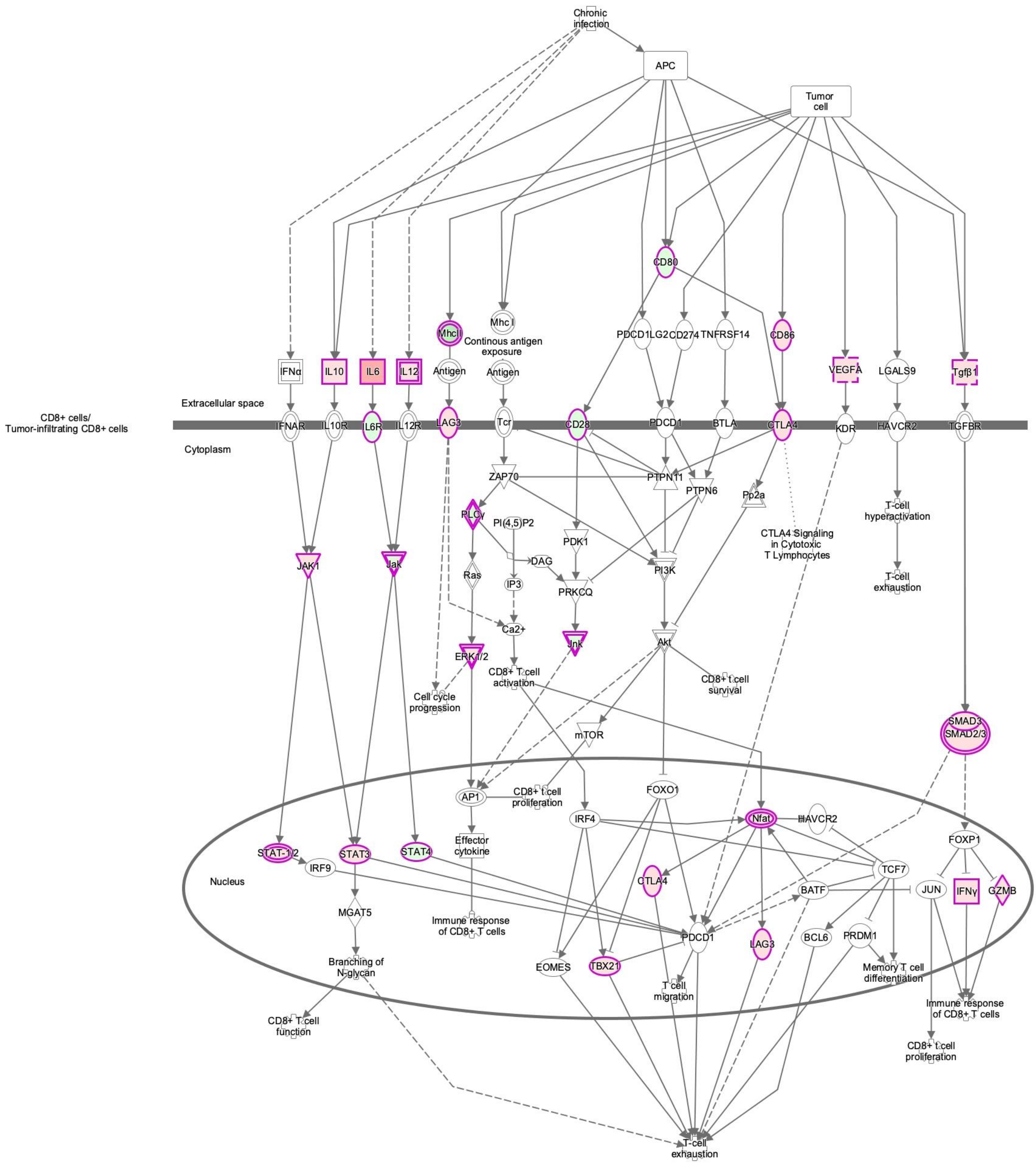

1.AA VDR/RXR activation (z-score = 2.33)

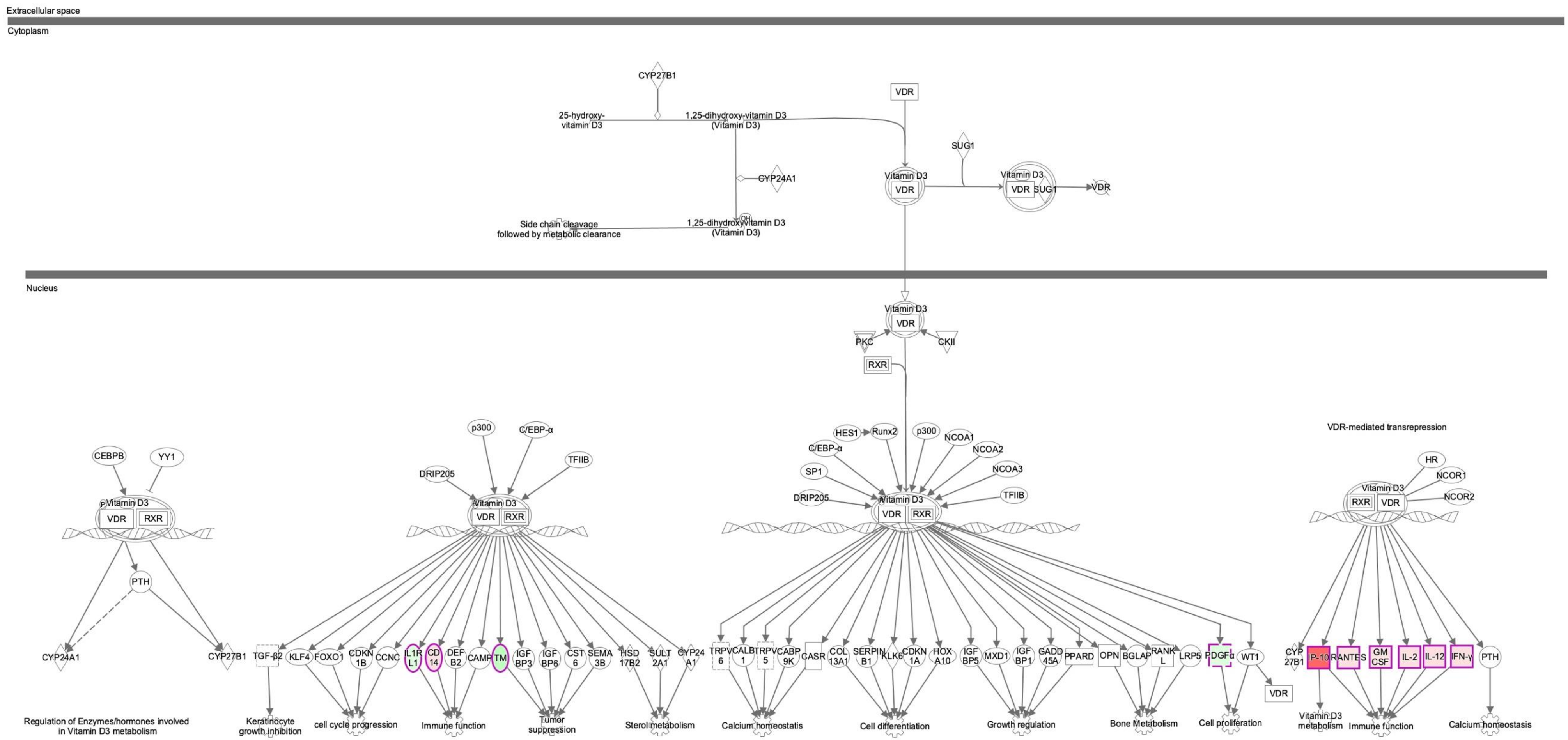

1.AB Role of IL17F in allergic inflammatory airway diseases (z-score = 2.31)

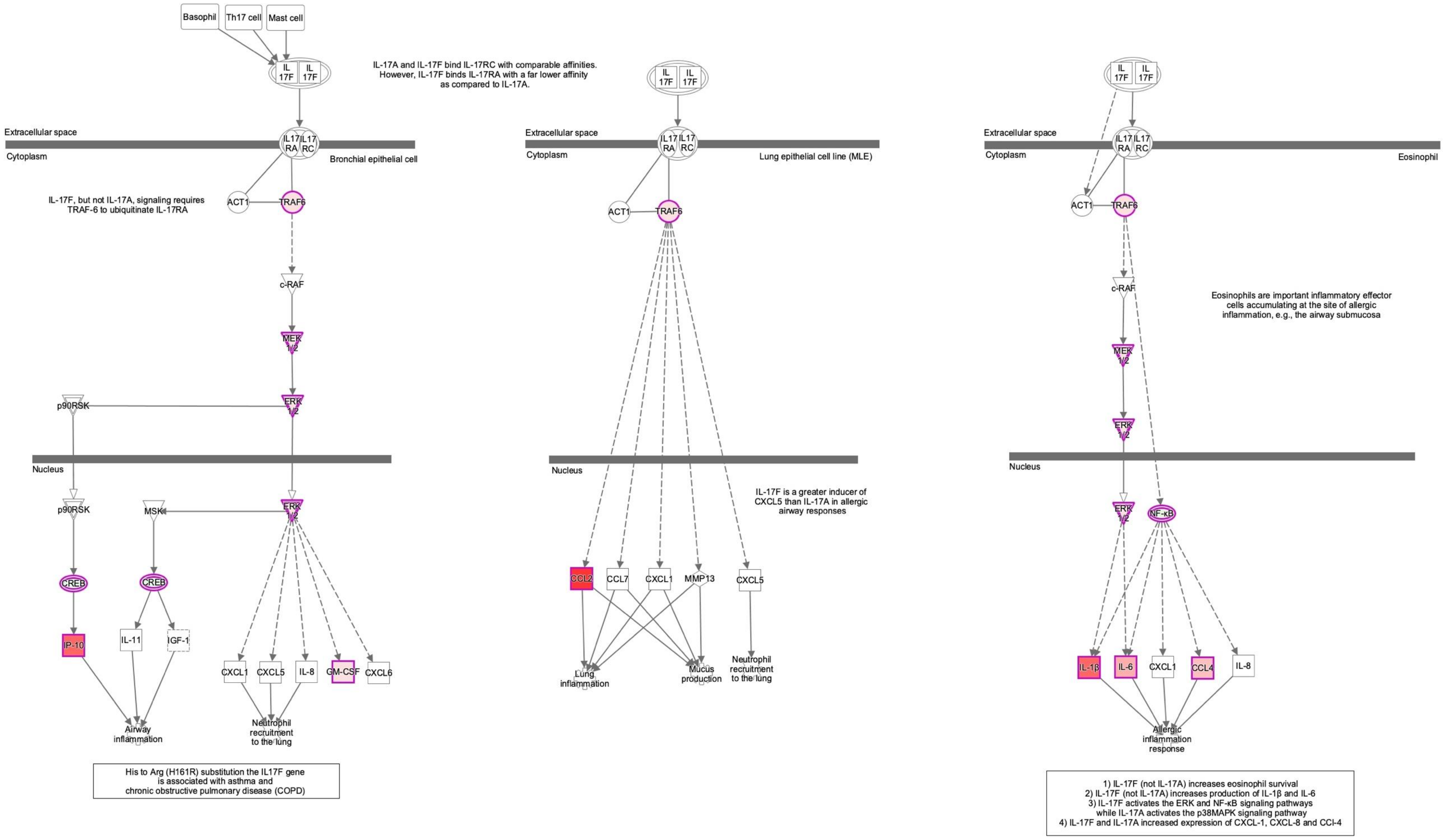

1.AC IL3 signaling (z-score = 2.31)

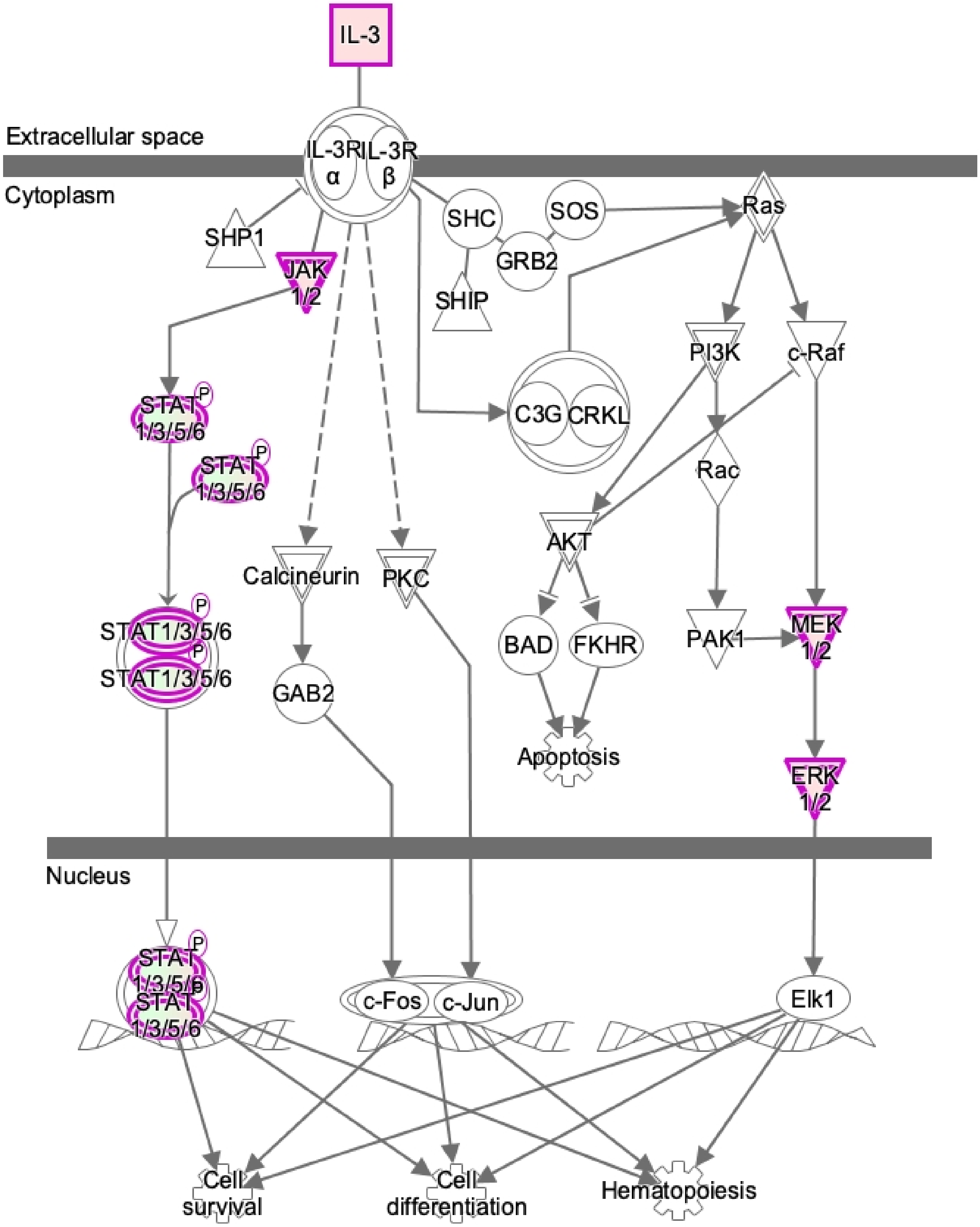

1.AD Telomerase signaling (z-score = 2.12)

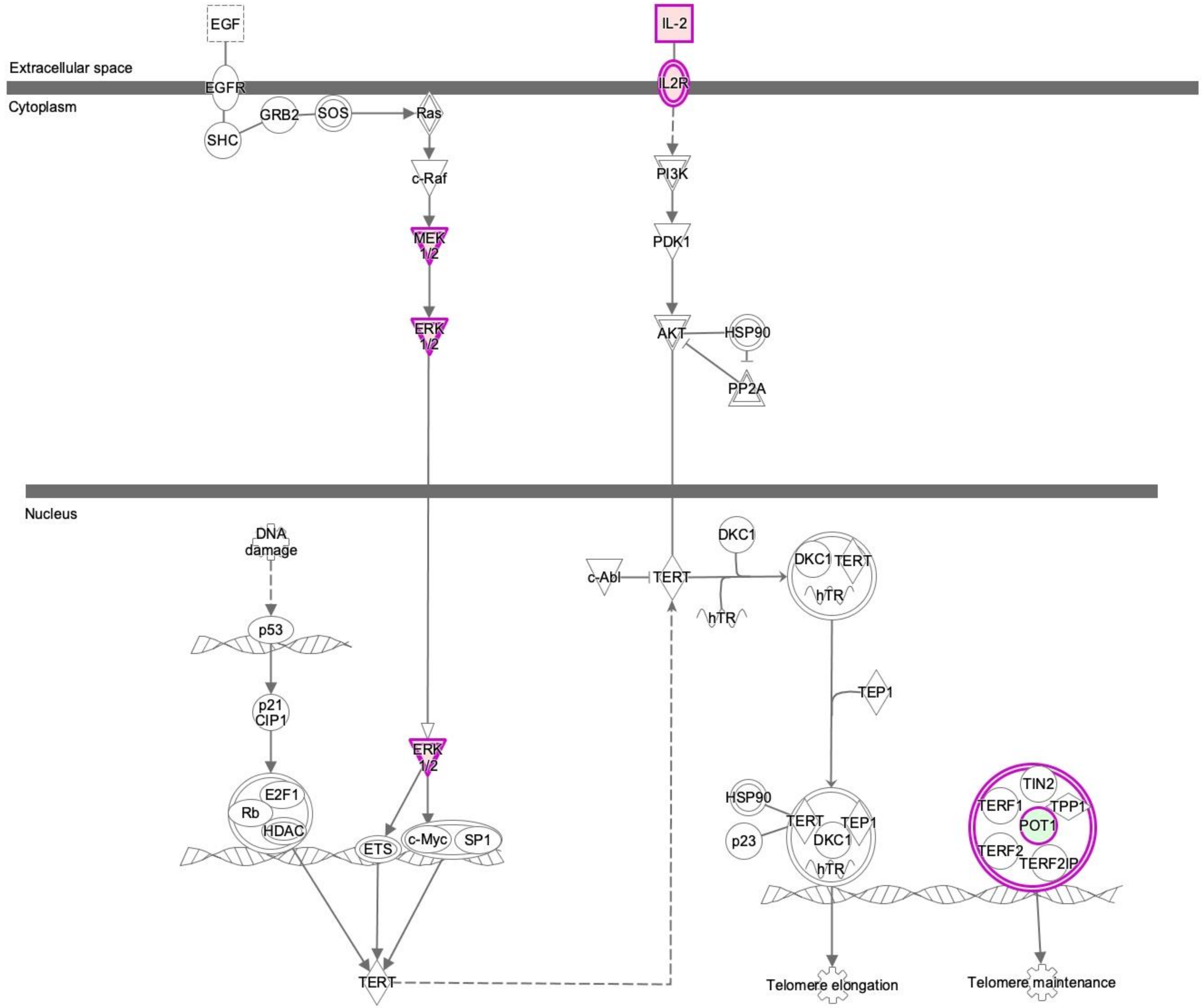

1.AE Interferon signaling (z-score = 2.12)

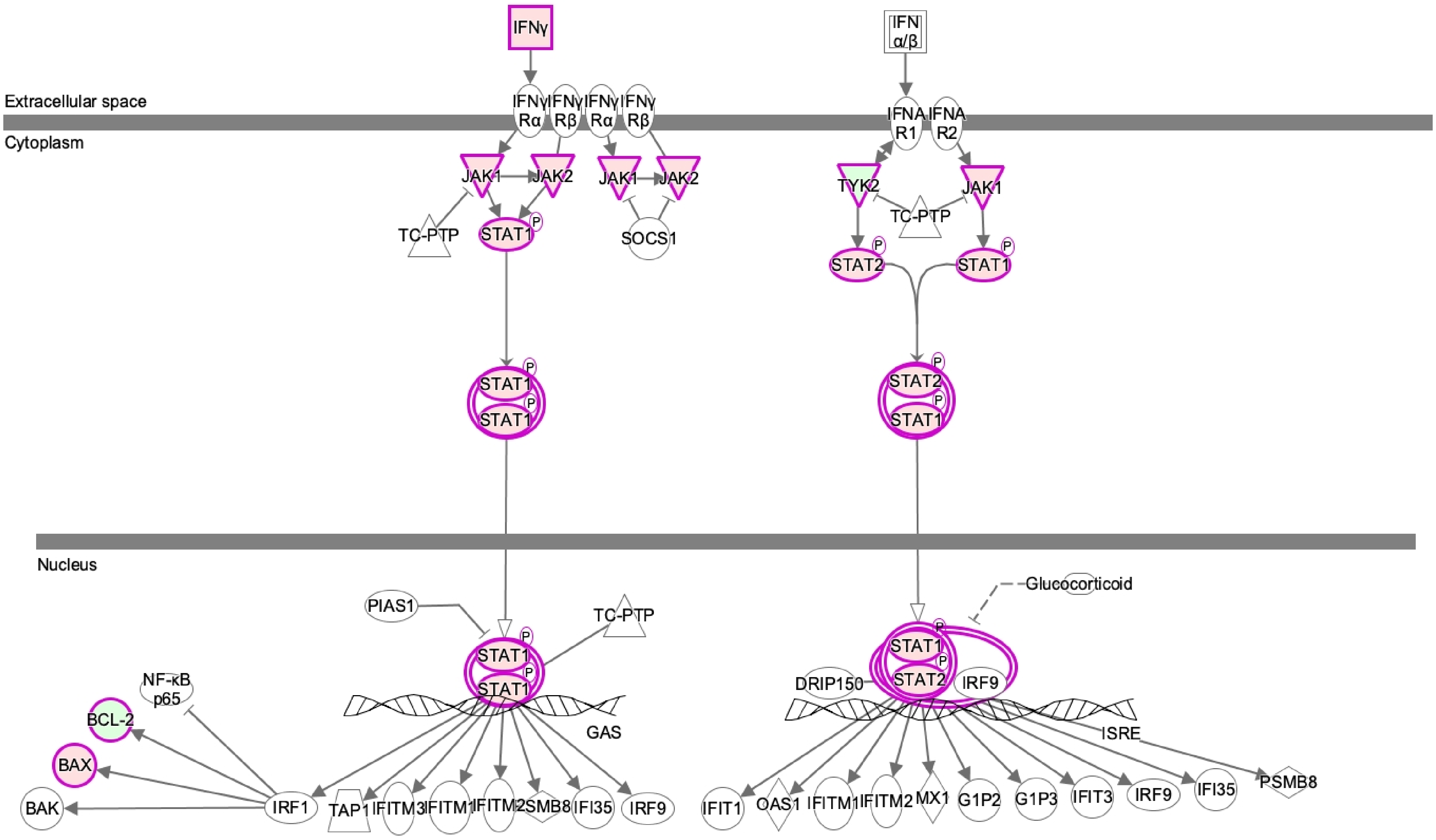

1.AF Th2 pathway (z-score = 2.08)

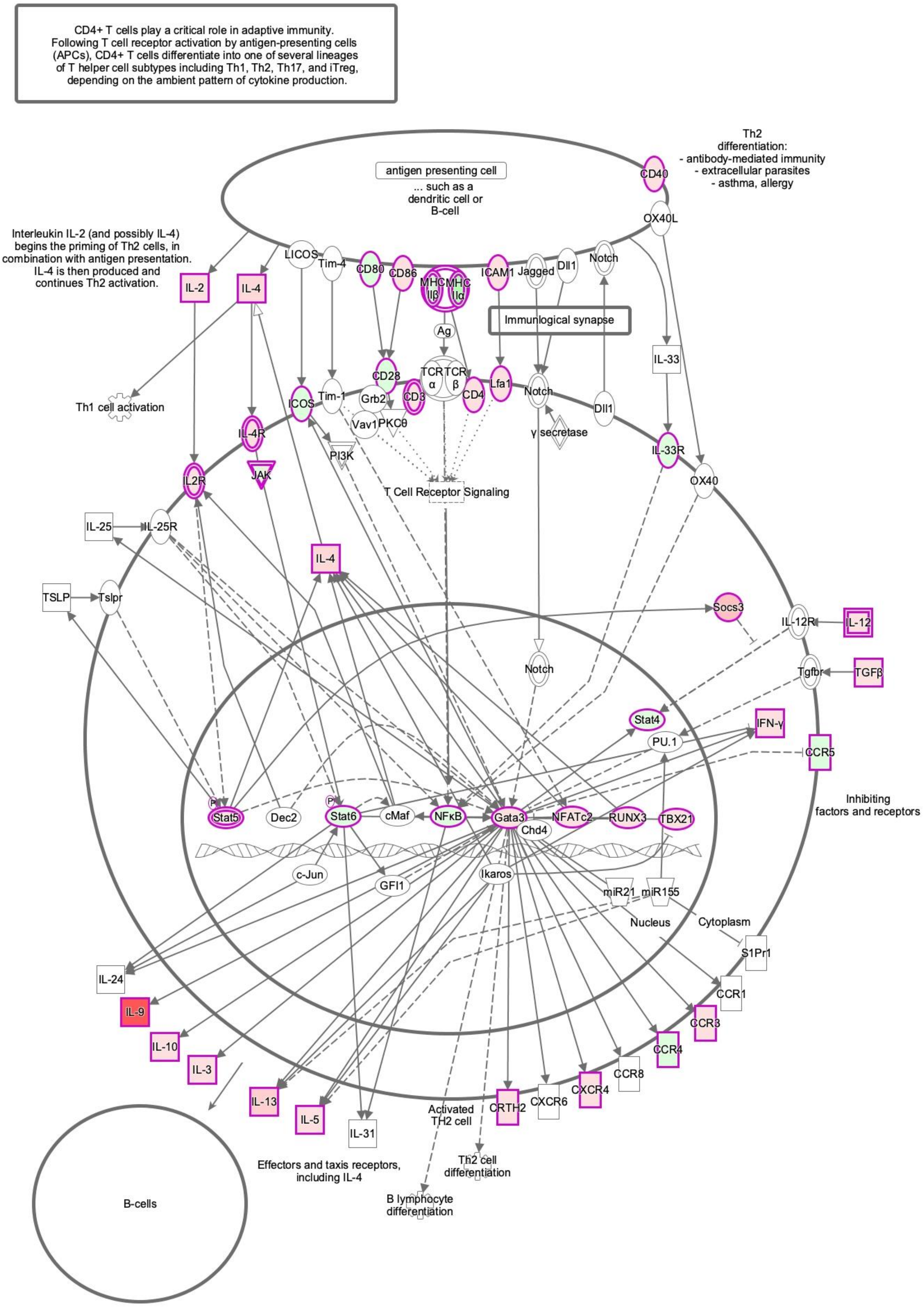

1.AG Wnt/Ca+ pathway (z-score = -2.11)

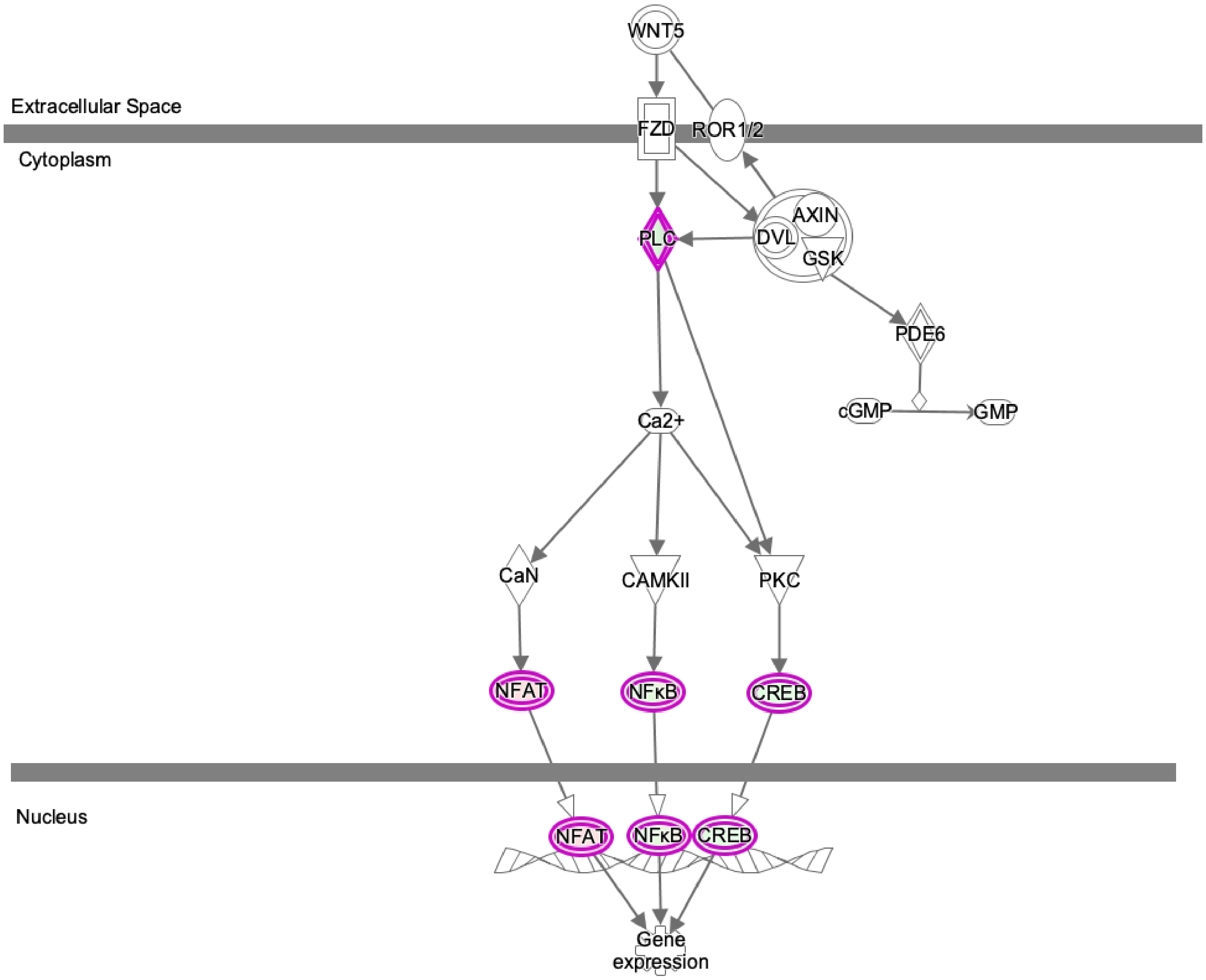

1.AH GPCR-mediated nutrient sensing in enteroendocrine cells (z-score = -2.11)

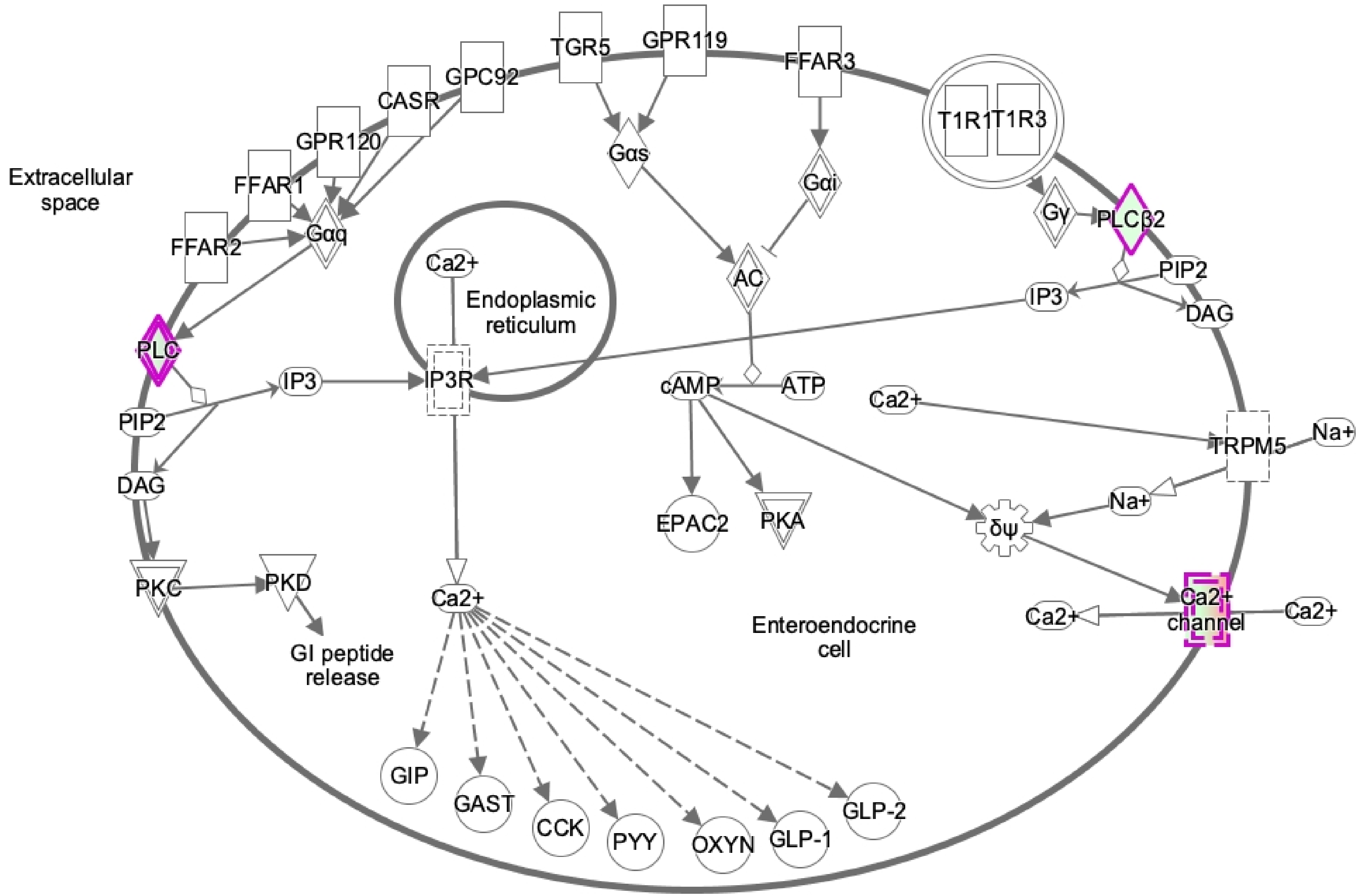

1.AI Dopamine-DARP31 feedback in cAMP signaling (z-score = -2.33)

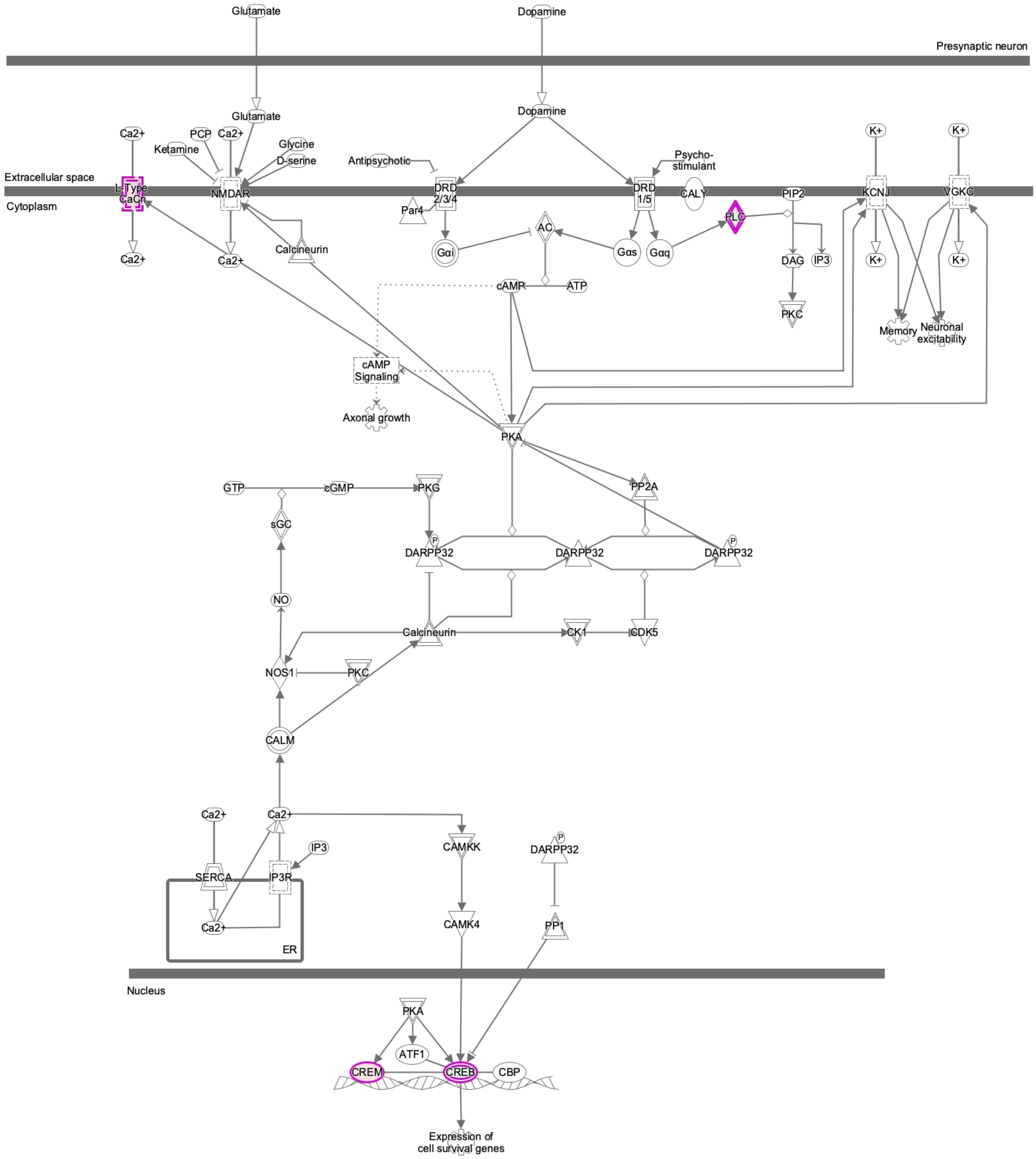

1.AJ D-myo-inositol (1,4,5)-triphosphate biosynthesis (z-score = -2.65)

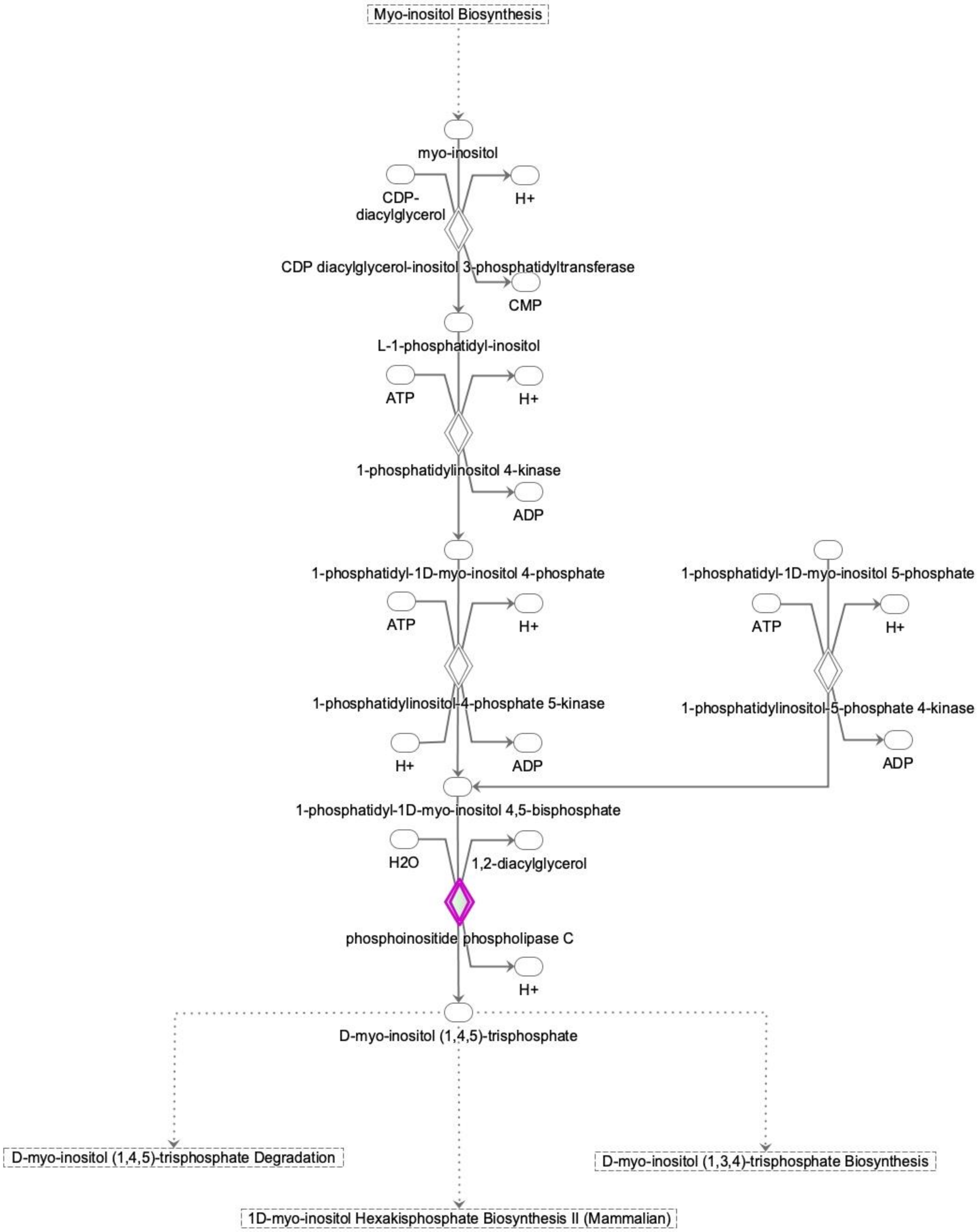

1.AK Sphingosine-1-phosphate signaling (z-score = -2.67)

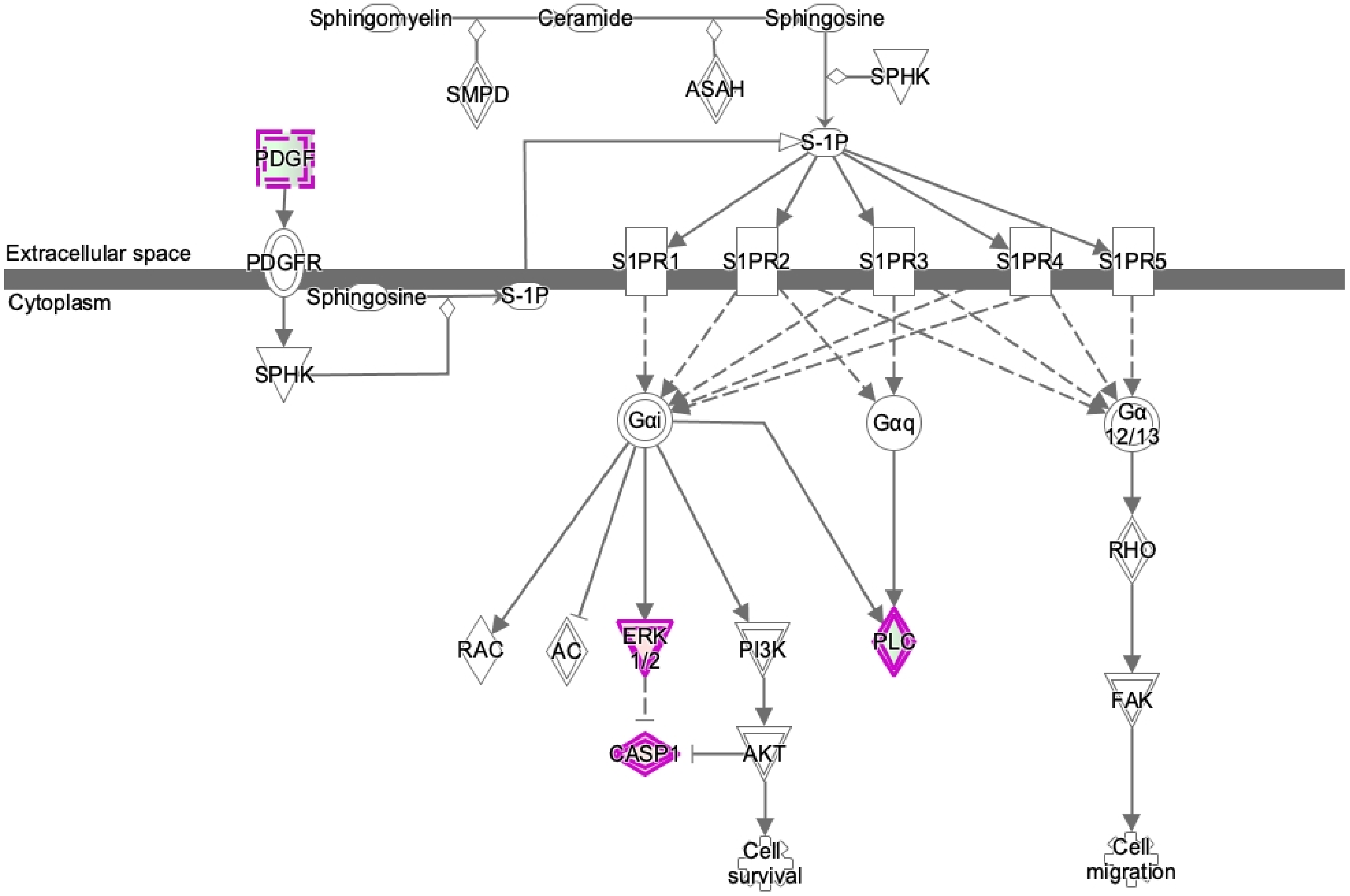

Supplement: Supplementary file 1 [file cells-11-00531-s001.zip › Liberman ea_SupplFig_Cells_V0.pdf]
