# Supplementary material for: Three Months of Strength Training Changes the Gene Expression of Inflammation-Related Genes in PBMC of Older Women: A Randomized Controlled Trial
Source: Cells. 2022 Feb 3;11(3):531. doi: 10.3390/cells11030531 (PMC8834561; doi:10.3390/cells11030531)
Supplement: Supplementary file 1 [file cells-11-00531-s001.zip › Liberman ea_SupplTable_Cells_V0.pdf]

Supplementary Table S1. Blood counts at baseline and after 3 months of resistance or flexibility training.

		IST percentile			SET percentile			CON percentile		
		median	25th	75th	median	25th	75th	median	25th	75th
White blood cells (10e3/uL)	Baseline	5.90	5.33	8.12	6.51	5.33	6.79	6.28	5.50	6.57
	3 months	5.60	4.85	6.99	6.60	5.27	6.95	6.59	5.42	8.50
	p-value	0.46			0.89			0.46		
Neutrophils (%)	Baseline	62.80	50.76	69.18	60.42	57.42	65.59	61.00	43.44	63.68
	3 months	55.32	48.36	67.78	60.78	48.66	66.73	54.06	40.16	66.15
	p-value	0.71			0.50			0.47		
Monocytes (%)	Baseline	8.39	7.92	10.08	5.45	4.71	6.88	7.82	5.58	8.00
	3 months	7.72	6.98	10.08	7.13	5.9	8.43	8.97	6.62	9.79
	p-value	0.72			0.22			0.07		
Eosinophils (%)	Baseline	1.43	0.6	1.99	2.77	0.79	4.01	4	1.81	5.38
	3 months	1.3	1.14	2.39	2.42	1.11	5.3	3.3	1.85	5.17
	p-value	0.58			0.69			0.46		
Basophils (%)	Baseline	0.37	0.27	0.5	0.58	0.39	0.8	0.79	0.56	1.41
	3 months	0.74	0.23	0.98	0.35	0.15	0.89	0.52	0.4	1.4
	p-value	0.14			0.5			0.27		
Lymphocytes (%)	Baseline	26.05	21.65	38.03	29.4	26.9	33.2	28.7	24.2	43.63
	3 months	33.55	22.35	41.08	29.8	25.1	37.5	34.2	21.68	45.75
	p-value	0.46			0.69			0.72		
CD3 cells (10e3/uL)	Baseline	1.06	0.88	1.21	1.02	0.79	1.53	0.93	0.76	1.47
	3 months	1.15	0.84	1.61	1.29	0.83	1.61	1.18	0.81	1.73
	p-value	0.13			0.50			1.00		
CD8+ T-cells (10e3/uL)	Baseline	0.44	0.32	0.60	0.29	0.26	0.43	0.29	0.15	0.45
	3 months	0.52	0.34	0.68	0.34	0.22	0.58	0.42	0.21	0.48
	p-value	0.72			0.50			0.50		
CD8+CD28+CD57- (naïve, %)	Baseline	38.10	18.95	63.48	29.80	28.05	50.80	70.10	46.90	78.10
	3 months	36.60	21.55	62.38	42.00	29.75	48.05	70.90	46.35	77.60
	p-value	0.72			0.50			0.50		
CD8+CD28-CD57- (memory, %)	Baseline	45.70	32.23	70.05	40.30	34.55	61.40	20.00	16.35	33.65
	3 months	54.55	28.15	75.18	52.20	35.95	56.80	21.50	19.65	35.80
	p-value	0.72			0.89			0.14		
CD8+CD57+ (%)	Baseline	5.05	4.30	22.23	16.10	5.35	26.80	5.20	3.05	24.45
	3 months	8.00	3.30	10.38	9.40	3.80	23.85	2.60	1.10	22.05
	p-value	1.00			0.50			0.23		
CD8+CD28-CD57+ (SPC, %)	Baseline	4.95	4.13	21.90	15.90	5.25	26.15	5.10	2.00	20.40
	3 months	7.70	3.28	10.10	9.20	3.60	23.75	1.70	1.10	18.90
	p-value	1.00			0.50			0.22		
CD8+CD28+CD57+ (SPC, %)	Baseline	0.15	0.10	0.35	0.20	0.10	0.65	1.10	0.55	4.05
	3 months	0.15	0.03	0.43	0.20	0.10	0.20	0.90	0.00	3.15
	p-value	1.00			0.46			0.13		

		IST			SET			CON		
			percentile			percentile			percentile	
		median	25th	75th	median	25th	75th	median	25th	75th
CD8- T-cells (10e3/uL)	*Baseline*	*0.61*	*0.55*	*0.62*	*0.77*	*0.45*	*1.16*	*0.76*	*0.51*	*1.06*
	3 months	*0.63*	*0.50*	*0.93*	*0.85*	*0.57*	*1.12*	*0.88*	*0.48*	*1.25*
	p-value	*0.47*			*0.50*			*1.00*		
CD8-CD28+CD57- (naïve, %)	*Baseline*	*93.85*	*86.73*	*96.63*	*94.40*	*89.60*	*96.50*	*99.20*	*91.90*	*99.70*
	3 months	*93.85*	*87.63*	*97.15*	*94.80*	*92.45*	*96.85*	*99.30*	*91.55*	*99.65*
	p-value	*0.29*			*0.69*			*0.85*		
CD8-CD28-CD57- (memory, %)	*Baseline*	*4.40*	*2.63*	*9.85*	*4.50*	*2.15*	*6.20*	*0.70*	*0.20*	*2.30*
	3 months	*4.80*	*1.88*	*10.65*	*4.50*	*1.95*	*6.30*	*0.50*	*0.20*	*3.00*
	p-value	*1.00*			*0.89*			*0.29*		
CD8-CD57+ (SPC, %)	*Baseline*	*2.05*	*0.45*	*3.43*	*1.90*	*0.65*	*4.40*	*0.20*	*0.05*	*5.70*
	3 months	*1.45*	*0.68*	*1.85*	*0.90*	*0.55*	*1.70*	*0.20*	*0.05*	*5.45*
	p-value	*0.47*			*0.18*			*0.66*		
CD8-CD28-CD57+ (SPC, %)	*Baseline*	*2.00*	*0.38*	*3.33*	*0.80*	*0.60*	*4.35*	*0.10*	*0.00*	*4.30*
	3 months	*1.35*	*0.58*	*1.75*	*0.90*	*0.30*	*1.45*	*0.10*	*0.00*	*4.05*
	p-value	*0.47*			*0.23*			*0.66*		
CD8-CD28+CD57+ (SPC, %)	*Baseline*	*0.10*	*0.03*	*0.10*	*0.10*	*0.00*	*0.60*	*0.10*	*0.05*	*1.40*
	3 months	*0.10*	*0.10*	*0.10*	*0.10*	*0.05*	*0.40*	*0.10*	*0.05*	*1.40*
	p-value	*0.32*			*0.66*			*1.00*		

IST = intensive strength training; SET = strength-endurance training, CON = control, SPC = senescence-prone cells; the values denote median (interquartile range); phenotype frequencies were expressed as percentages within the CD3+CD8+ or CD3+CD8− T-cells. P-values based on Wilcoxon Signed Rank test
